# A systematic review of validity of US survey measures for assessing substance use and substance use disorders

**DOI:** 10.1186/s13643-024-02536-x

**Published:** 2024-06-27

**Authors:** Yuni Tang, Erin Caswell, Rowida Mohamed, Natalie Wilson, Edis Osmanovic, Gordon Smith, Summer D. Hartley, Ruchi Bhandari

**Affiliations:** 1 Department of Epidemiology and Biostatistics, School of Public Health, West Virginia University, Morgantown, WV 26501 USA; 2 Department of Pharmaceutical Systems and Policy, School of Pharmacy, West Virginia University, Morgantown, WV USA; 3 Health Affairs Institute, West Virginia University, Morgantown, WV USA; 4 Present Address: Highway Safety Research Center, University of North Carolina at Chapel Hill, Chapel Hill, NC USA; 5 Present Address: Biological Sciences Division, University of Chicago, Chicago, USA IL; 6 Hartley and Associates, WV Morgantown, USA

**Keywords:** Systematic review, Validity testing, Substance use, Measures

## Abstract

**Background:**

The steep rise in substance use and substance use disorder (SUD) shows an urgency to assess its prevalence using valid measures. This systematic review summarizes the validity of measures to assess the prevalence of substance use and SUD in the US estimated in population and sub-population-based surveys.

**Methods:**

A literature search was performed using nine online databases. Studies were included in the review if they were published in English and tested the validity of substance use and SUD measures among US adults at the general or sub-population level. Independent reviews were conducted by the authors to complete data synthesis and assess the risk of bias.

**Results:**

Overall, 46 studies validating substance use/SUD (*n* = 46) measures were included in this review, in which 63% were conducted in clinical settings and 89% assessed the validity of SUD measures. Among the studies that assessed SUD screening measures, 78% examined a generic SUD measure, and the rest screened for specific disorders. Almost every study used a different survey measure. Overall, sensitivity and specificity tests were conducted in over a third of the studies for validation, and 10 studies used receiver operating characteristics curve.

**Conclusion:**

Findings suggest a lack of standardized methods in surveys measuring and reporting prevalence of substance use/SUD among US adults. It highlights a critical need to develop short measures for assessing SUD that do not require lengthy, time-consuming data collection that would be difficult to incorporate into population-based surveys assessing a multitude of health dimensions.

**Systematic review registration:**

PROSPERO CRD42022298280.

**Supplementary Information:**

The online version contains supplementary material available at 10.1186/s13643-024-02536-x.

## Introduction

Substance use remains a serious adverse health risk in the United States (US). Forty million Americans reported illicit drug use in the past month in 2021, among people aged 12 years or older (Substance Abuse and Mental Health Services Administration, 2022b), with over 106,000 people in the US fatally overdosing in 2021 (National Institute on Drug Abuse, 2023). This is a dramatic increase of approximately 15% in overdoses within 1 year, signifying critical, life-threatening substance use problems and an associated overdose epidemic throughout the county. Notably, substance use problems that met the criteria for a substance use disorder (SUD) were reported by a sizeable proportion of the US population. More than 46 million people aged 12 years or older met the *Diagnostic Statistical Manual of Mental Disorders* (DSM-V) criteria for SUD in the past year, according to the National Survey of Drug Use and Health (NSDUH), with the highest percentage of people with SUD being young adults aged 18–25 (25.6%), followed by adults aged 26 or older (16.1%) [1]. Unfortunately, population-based assessments for SUD are rare beyond the NSDUH, especially at substate levels, although imperative to inform appropriate resource allocation and population-based interventions for states responding to the SUD and overdose epidemics.

There are few population-based surveys conducted in the US that assess substance use and/or SUD. NSDUH is a good example of a survey that monitors annual national trends in substance use and mental health issues in the US and provides estimates of the need for substance use prevention and treatment programs [2]. However, it involves lengthy questions and branching logic that are not feasible for use in surveys covering multiple health domains. Another validated tool to assess SUD is the National Addictions Vigilance Intervention and Prevention Program (NAVIPPRO™) Addiction Severity Index-Multimedia Version® (ASI-MV®) [3]. However, results from this measure may not be generalizable because it is only used to evaluate those already seeking SUD treatment. In addition, selection bias is likely because the participants are selected based on convenience sampling among treatment centers [4]. Other measures that have been validated for assessing substance use in the US are Drug Abuse Screening Test (DAST) [5], Alcohol, Smoking, and Substance involvement Screening Test (ASSIST), and tobacco, alcohol, prescription medication, and other substance use (TAPS) [6]. However, these survey measures also require multiple, lengthy questions to estimate the prevalence of SUD.

Validated substance use and SUD measures that are shorter and more versatile are needed to ease the incorporation of these measures into more multidimensional population health surveys to better assess and respond to the current US substance use and overdose epidemics. Much work has been done on validating alcohol and tobacco measures, such as ASSIST and TAPS [6]. We know of no review of validation research conducted on other substance use and/or SUD measures among the US population, although previous studies provide valuable insights into measures assessing the efficacy of substance use measures and interventions [7] and addressing psychometric properties of screening tools among specific settings or populations [8]. Thus, the purpose of this review is to comprehensively summarize published literature investigating the validity of substance use and SUD measures, other than alcohol and tobacco use, in US surveys to advance the use of these validated measures on more population-based surveys.

## Methods

### Search strategy

This systematic review has followed the Preferred Reporting Items for Systematic reviews and Meta-analyses (PRISMA) guidelines [9] and was registered through PROSPERO (CRD42022298280). Potential eligible studies were identified by using the following nine electronic databases, starting from their inception up to November 22, 2021: PubMed, Scopus, CINAHL, PsycINFO, Academic Search Complete, Web of Science, ProQuest Theses and Dissertation Global, and Google Scholar. Primary keywords and phrases used for searching included “healthcare survey,” “mental health,” “substance use,” and “validity.” Detailed search strategies corresponding to the specific databases are shown in Supplementary Table 1.

The following study inclusion criteria were established a priori for use in this systematic review: [1] Utilized existing surveys or questionnaires at the county level or higher (validation may have been done at a sub-population level) or at clinical settings in the US; [2] to ensure the reviewed measures are applicable to US populations, and only studies conducted in the US were included in this review; [3] validity/validation testing conducted for measures of mental health and/or substance use; [4] study sample consisted of adults 18 years of age or older; [5] studies published in English language; and [6] peer-reviewed, published studies, official reports from surveys, and doctoral dissertations. In addition, exclusion criteria were applied to those studies that [1] assessed the validity of measures unrelated to mental health/substance use (i.e., physical activity, chronic disease, infectious disease); [2] assessed the validity of alcohol and/or tobacco measures only; [3] were published as abstract only or did not have full texts available; [4] were protocols, editorials, reviews, or commentary; [5] validated language translation or cultural version of an instrument; and [6] were conducted internationally. In order to better align with the aims of this review, studies validating only alcohol/tobacco use measures were excluded because they have been widely studied in previous literature [10–13].

### Quality assessment

An adapted risk-of-bias tool was developed for the purpose of this systematic review to assess the validity of substance use and mental health survey instruments. This methodological quality assessment tool was adapted from a previously published tool which evaluated the rigor of validity testing in the Behavioral Risk Factor Surveillance System (BRFSS) literature [14]. The new risk-of-bias tool was used to assess the quality of the [1] methodology and [2] statistical analyses of studies included in the systematic review. The methodological component was scored from 0 to 3 (3 = studies utilizing a physical measurement(s) as a comparator during validity testing, which were considered to be the “gold standard,” 2 = studies using measures other than actual physical measure, 1 = studies that conducted face validity based on the researcher’s judgment or a collective judgment, 0 = studies that did not report on the measurement used for validity testing). The statistical analysis component was scored from 0 to 2 (2 = using statistical analyses such as sensitivity and specificity, correlation coefficient, or mean difference, 1 = reporting prevalence estimates only, 0 = no information on statistical analysis was reported). The methodological and statistical component scores were then totaled for an overall quality assessment score. Total scores ranged from 0 to 5, with 5 demonstrating the highest quality.

### Data synthesis

All identified studies were imported to an EndNote library. After removing duplicates, the initial title and abstract screenings were conducted independently by three reviewers (Y. T., N. W., E. O.) using the pre-established inclusion and exclusion criteria. It was followed by the full-text review conducted independently by three reviewers (Y. T., E. C., R. M.) for the first 10% of the included studies. They then convened to review their selections to ensure agreement and refine criteria. Inter-rater reliability was calculated in STATA [15] using the Gwet’s AC to ensure agreement [16]. The remaining 90% of the selected articles were then split between the three reviewers for full-text review. Articles where a reviewer was not sure if they should be included or excluded were discussed among the three reviewers and decided by the senior author for final selection.

A data extraction form was created in Microsoft Excel to facilitate data extraction and synthesis. The form could capture up to 46 variables for each study. These variables were grouped into four main categories: study characteristics (authors, reference, year of publication, and name of journal), measure characteristics (whether the measure was used for disorder screening, the SUD being assessed by the measure, response rate, study duration, items measured, recall period, and recruitment procedure), participant characteristics (overall health status, age, sex, race, income, education), and validation methods (type of validation, statistical analysis, comparison measure, and key results). Additionally, a single article could be considered as multiple studies if it validated measures among multiple study populations. Articles that validated multiple survey measures among the same study population were considered to be one study. We evaluated the different types of validity using pre-established definitions to standardize the understanding of validity among reviewers. Our focus was on examining criterion validity (including concurrent, predictive, and content validity) and construct validity (encompassing convergent, discriminant, and factorial validity). Specifically, criterion validity was examined through comparisons with “gold standard” measures where available or through the use of clinically established diagnostic criteria and outcomes. Face validity was determined if the article could demonstrate the extent to which a substance use measured what it intended to measure. Lastly, construct validity was assessed through statistical analyses examining the correlation between survey measures and related constructs, thus ensuring that measures accurately reflect the theoretical components of substance use and SUDs. Articles that did not specify the validation methods were discussed among the three reviewers and decided by the senior author for consensus if discrepancies existed.

All data were coded independently by two reviewers (Y. T., E. C.). After extracting data from the first 10 articles, the two reviewers met to discuss any discrepancies among coding strategies. Disagreements were brought to the senior author (R. B.) for conflict resolution. Although the inclusion and exclusion criteria were determined a priori, the completion of data extraction demonstrated unique differences present between mental health and substance use studies that evaluated the psychometric properties of their respective measures. As the study developed, the results gathered from the data synthesis for substance use were substantially different from mental health assessment, and the authors determined that these separate domains would be better discussed in two separate manuscripts. Thus, the results presented in this study are from studies that validated substance use measures identified in our search.

## Results

### Study characteristics

**Fig. 1:**
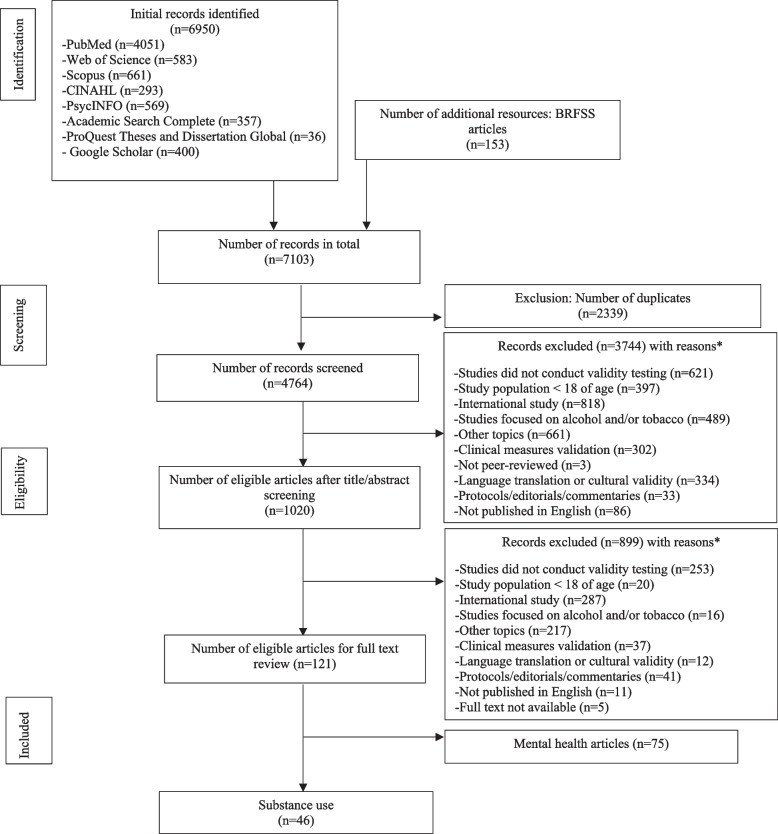
Flow chart for the selection of studies*. *Studies could have been excluded for multiple reasons

A total of 6950 results were initially obtained from the search. An additional 153 articles were identified by reviewing BRFSS reference lists [17]. A flow diagram documenting the search process and reasons for excluding studies is shown in Fig. 1. Of the 7103 articles, 2339 were duplicates and were excluded before the abstract/title review. After reviewing 4764 abstracts/titles, 3744 articles were excluded. Of the 1020 articles, a full-text review of the first 10% of articles demonstrated an almost perfect inter-rater reliability agreement between reviewers on which articles met the inclusion criteria (Gwet’s AC: 0.8517 (0.8000–1.0000)). Following review of the full article text, 899 articles were removed. The key reasons for excluding the articles were because they [1] did not conduct validity testing (*n* = 874), [2] were conducted outside the United States (*n* = 1105), or [3] were focused on topics other than substance use (*n* = 878). For this review, a total of 46 articles met the inclusion criteria (Fig. 1). The characteristics of those 46 selected studies are presented in Table 1.

**Table 1:** Characteristics of included studies of validation testing

First author and publication year	Participant’s characteristics	Study characteristics	Survey instrument/questionnaire characteristics	Validation methods	Key findings	ROB assessment
Alexander and Leung [18]	• Aged ranged from 18 to 54 with mean age of 33.2 • Most of participants were male (*n* = 64, 58.7%) • 63% White, 20% Black, and 17% Hispanic	Non-population-based study evaluated the concurrent, convergent, and discriminant validity between the MSI-X and five other instruments in the alcohol and drug program	MSI-X • Specific substance use: Marijuana use • Sample size: *n* = 107 • Response rate = 13.4% • Survey duration: September 2001–June 2002	Construct (convergent) validity • Comparison measure: Compared with SASSI-3 overall “decision rule” for those most likely to be diagnosed with substance dependence or substance abuse • Statistical analysis: Two separate independent sample *t*-tests Criterion (concurrent) validity • Comparison measure: Comparing with DSM-G-MI, DAST-20, SASSI-3, and ASI interviewer severity rating scales and selected variables • Statistical analysis: Correlation coefficients	Those who had a high probability of substance dependence had a significantly (*t* = 3.256, *df* = 43.388, *p* = .002) higher number of MSI-X problems (mean = 6.2) than those who had a low probability for substance dependency (mean = 2.4). The concurrent validity analysis reveals excellent, strong significant positive association between MSI-X scores and the DSM-G-MI (*r* = 0.811; *p* < .001), good to moderately strong significant positive associations with the DAST-20 scores (*r* = 0.531; *p* < .001), and two specific SASSI-3 subscale scores, the Face Valid Other Drugs (FVOD) subscale scores (*r* = 0.626; *p* < .001) and correctional (COR) subscale (*r* = 0.522; *p* < .001)	5
Appleby, Dyson [19]	• Mean age of 32 years	Non-population-based study conducted in the inpatient clinical setting and used CUAD by comparing CUAD severity ratings with severity ratings from the other instruments used in the study and by determining its sensitivity and specificity in relation to SCID diagnoses of substance use disorders	CUAD • Specific substance use: Not reported • Sample size: 100 • Response rate: Not reported • Survey duration: Not reported	Construct validity • Comparison measure: Comparing CUAD severity ratings with severity ratings from other instruments used in the study: SMAST, DAST, ASI • Statistical analysis: Chi-square statistical analysis Criterion validity (concurrent validity) • Comparison measure: Comparing CUAD severity ratings with severity ratings from ASI alcohol and drug scales • Statistical analysis: Sensitivity	This study showed a highly significant positive correlation between the CUAD alcohol subscale and the SMAST and an even stronger association between the CUAD drug subscale and the DAST. Additionally, high positive correlations between CUAD and ASI alcohol and drug scales and both current substance use measures provided additional evidence of concurrent validity. Seventy-three patients had a SCID lifetime diagnosis of an alcohol use disorder, and the CUAD correctly identified 51 of them (70% sensitivity), which also showed evidence criterion validity	4
Boothroyd, Peters [20]	• Mean age of 40.1 (*SD* = 12.2) • 25.4% were males, and 74.6% were females	Population-based study examined the SSI’s psychometric properties within Medicaid recipients in Florida	SSI-SA • Specific substance use: Not reported • Sample size: *n* = 6664 • Response rate: 35–45% • Survey duration: 1998–2008	Construct validity (convergent validity) • Comparison measures: Examine the association of respondents’ SSI-SA classification at cutoff of 4 vs. at or above the cutoff 4 with their scores on a measure of general functioning, an indicator of current substance use, and quality of life • Statistical analysis: Independent *t*-tests sensitivity, specificity, PPV, NPV, ROC curve analysis	The SSI-SA had excellent internal consistency (0.85). Evidence of the SSI’s validity was strong. Using the recommended SSI-SA cutoff score of 4 or higher to indicate the presence of a substance abuse problem, the SSI-SA had respectable sensitivity (0.82) and specificity (0.90)	4
Broderick, Richmond [21]	• Mean age (SD) = 43 (15) years • Most of participants were males (57% vs. 43%) • Hispanic (37%), White (41%), and Black (18%)	Non-population-based study conducted in the inpatient clinical setting to evaluate two brief screen questions to assess the degree to which these single-item screening questions detected risky substance use compared to a longer, validated screening tool	Two-item brief screen (survey name not reported) • Specific substance use: Illicit drugs and marijuana • Sample size: *n* = 1218 • Response rate: *n* = 72% • Survey duration: August 25–October 31, 2010	Construct validity • Comparison measure: ASSIST • Statistical analysis: Sensitivity and specificity	Sensitivity values for the marijuana and street drug questions were 72% and 40%, respectively. Specificity values for the marijuana and street drug questions were 96% and 99%, respectively	5
Carter, Yu [6]	• Mean age (SD) = 49 (14.9) • Most participants were female (61.9%) • White (92.5%), Black/African American (4.5%), and Hispanic (0.9%)	Non-population-based study conducted in the community inpatient clinical setting to validate the performance of TAPS tool compared to a reference-standard substance use assessment	TAPS • Specific substance use: Not reported • Sample size: *n* = 1523 • Response rate: Not reported • Survey duration: November 2019 to October 2020	Construct validity • Comparison measure: ASSIST • Statistical analysis: AUC of ROC curve	The TAPS tool showed fair or better discrimination between moderate risk use and high-risk use for tobacco, alcohol, and prescription opioids (*AUCs*: 0.75–0.97) and fair or better discrimination between low-risk and moderate-risk use in five of eight subscales, including tobacco, alcohol, marijuana, stimulants, and heroin (*AUCs*: 0.70–0.92)	5
Chasnoff, Wells [22]	All pregnant women 18 years of age or older	Non-population-based study conducted in the inpatient clinical setting to validate the 4P’s Plus to identify women whose substance-use levels fall below the DSM-IV criteria but still at risk from any level of use of alcohol or illicit drugs	4P’s Plus • Specific substance use: Not reported • Sample size: *n* = 228 • Response rate: 59% • Survey duration: Not reported	Criterion validity (predictive validity) • Comparison measure: Comparing the 4P’s Plus positive and negative screens with positive and negative clinical assessment • Statistical analysis: Sensitivity, specificity, PPV, NPV	The overall reliability for the 5-item measure was 0.62. Sensitivity and specificity were very good at 87 and 76%, respectively. Positive predictive validity was low (36%), but negative predictive validity was quite high (97%). Of the 31 women who had a positive clinical assessment, 45% were using less than 1 day per week	5
Dennis and Davis [23]	• Mean age (SD) = 31 (11) years • 59% of the study population were males, and 41% were females • White: 64%, African American/Black: 10%, and Other race: 10%	Non-population-based study conducted in the outpatient clinical setting to examine the psychometric properties of core GAIN-Q3 assessment	GAIN-03 • Specific substance use: Not reported • Sample size: *n* = 10,167 • Response rate: Not reported • Survey duration: 2002–2010	Construct validity (convergent and discriminant validity) • Comparison measure: Compared the correlation between the screener and the full GAIN-I scale • Statistical analysis: Pearson correlation matrix between the shortened screener and the full-length scores, ROC analysis	Despite the condensed lengths of the screening measures compared with their longer versions, the reliability estimates are within the good to excellent range (0.7 to 0.9) in terms of internal consistency for 7 of the 10 screeners for adults. Moreover, there is strong evidence for the measures’ convergent and discriminant validity and efficiency (i.e., maximum information gathered in as few items possible) relative to the full-length scales as well as relative to other scales in the full GAIN-I	5
Dezman, Gorelick [24]	• All participants aged over 18 years old • Most participants were male (*n* = 803, 72.0%) • Caucasian: *n* = 627 (56.2%), African American: *n* = 481 (43.1%), Hispanic: *n* = 7 (0.6%)	Non-population-based study conducted in the trauma inpatient clinical setting to test characteristics of a 4-item drug CAGE questionnaire to detect DUDs	CAGE • Specific substance use: Not reported • Sample size: *n* = 1115 • Response rate: 58% • Survey duration: September 1994–November 1996	Criterion validity • Comparison measure: Comparison with SCID-generated DUD diagnoses as the standard • Statistical analysis: Sensitivity, specificity, PPV, NPV, and AUC	The drug CAGE screen had an *AUC* = 0.9. Each individual question had a high AUC (0.78–0.87). The drug CAGE questionnaire had high AUCs across all sociodemographic and injury mechanism subgroups, both for each individual question and overall	5
Duncan, Sacks [25]	The study showed self-reported demographic characteristics of the study sample composed of African American, Latinos, and Whites. The majority of the sample in each racial and ethnic group was male. The average age was 36.03 years for African Americans, 32.74 years for Latinos, and 36.02 years for Whites. Males: 56.03% for African Americans, 60.8% for Latinos, and 36.02% for Whites	Non-population-based study conducted in the prison substance abuse treatment programs to test the stability of the performance of the CODSI-MD and SMD across three racial/ethnic groups of offenders entering prison substance abuse treatment programs	CODSI-MD • Specific substance use: Not reported • Sample size: *n* = 353 • Response rate: Not reported • Survey duration: Not reported	Criterion validity • Comparison measure: Compare three racial or ethnic groups of offenders entering prison substance abuse treatment programs • Statistical analysis: Sensitivity, specificity, PPV, NPV, positive and negative likelihood ratio	No statistical differences in sensitivity or specificity for either CODSI-MD or SMD across the African American, Latino, and White prisoner groups	4
			CODSI-SMD • Specific substance use: Not reported • Sample size: *n* = 353 • Response rate: Not reported • Survey duration: Not reported	Criterion validity • Comparison measure: Compare three racial or ethnic groups of offenders entering prison substance abuse treatment programs • Statistical analysis: Sensitivity, specificity, PPV, NPV, positive and negative likelihood ratio		
Han, Sherman [26]	• Mean age (SD) = 47.1 (13.4) years • Most participants were males (72.0%) • White: 56.2%, African American/Black: 40.8%, American Indian/Alaska Native: 1.8%, Asian: 2.5%, Other race: 22.1%	Non-population-based study conducted in the inpatient clinical setting to assess the diagnostic accuracy of the SUBS in comparison to ASSIST and AUDIT-C to identify unhealthy and high-risk alcohol and drug use among hospitalized current smokers	SUBS • Specific substance use: Not reported • Sample size: *n* = 439 • Response rate: Not reported • Survey duration: Not reported	Criterion validity (concurrent) • Comparison measure: AUDIT-C for alcohol use and ASSIST for drug use • Statistical analysis: ROC analysis, sensitivity, specificity	The SUBS had a sensitivity of 98% (95% *CI* 95–100%) and specificity of 61% (95% *CI* 55–67%) for unhealthy alcohol use, a sensitivity of 85% (95% *CI* 80–90%) and specificity of 75% (95% *CI* 78–87%) for illicit drug use, and a sensitivity of 73% (95% *CI* 61–83%) and specificity of 83% (95% *CI* 78–87%) for prescription drug nonmedical use. For identifying high-risk use, a higher cutoff (response of “3 or more days” of use indicates a positive screen), the SUBS retained high sensitivity (77–90%), and specificity was 62–88%	4
Harris, Ellerbe [27]	Not reported	Non-population-based study conducted in the inpatient and outpatient clinical setting to assess the specification validity of the 2009 HEDIS substance use disorder initiation and engagement	HEDIS • Specific substance use: Not reported • Sample size: *n* = 2726 • Response rate: Not reported • Survey duration: Not reported	Criterion validity (specification validity) • Comparison measure: Not reported • Statistical analysis: PPV	The PPV were excellent (> 9 0%) for residential and outpatient records selected from addiction treatment programs but more modest for records generated in non-addiction settings and were highly variable across facilities	1
Hasin, Greenstein [28]	• More participants were females than males (54.5% vs. 45.5%) • White: 67.8%, African American/Black: 22.5%, American Indian/Alaska Native: 2.1%, Asian: 0.7%, and Other race: 6.9%	Population-based study used a test–retest design to compare concordance of the NESARC-III survey questions with a semi-structured interview, the Psychiatric Research Interview for Substance and Mental Disorders, DSM-5 version (PRISM-5) administered by a clinician	AUDADIS • Specific substance use: Not reported • Sample size: *n* = 712 • Response rate: 92.5% • Survey duration: June 2012–July 2013	Criterion validity (concurrent validity) • Comparison measure: Physician diagnosis • Statistical analysis: Intraclass correlation coefficients	Concordance of the AUDADIS-5 and the PRISM-5 for DSM-5 diagnoses of substance use disorders ranged from fair to good (*κ* = 0.40–0.72). Concordance on dimensional scales was excellent (*ICC* ≥ 0.75) for the majority of DSM-5 SUD diagnoses and fair to good (*ICC* = 0.43–0.72) for most of the rest	5
Hasin, Keyes [29]	For frequent marijuana users: 66.9% males and 33.1 females, 75.4% White, 11.4% African American/Black, 3.7% American Indian/Alaska Native, 2.2% Native Hawaiian or Other Pacific Islander For marijuana use only users: 58.2% males and 41.8% females, 71.1% White, 15.3% African American/Black, 2.7% American Indian/Alaska Native, 2.7% Native Hawaiian or Other Pacific Islander	Sub-population-based study and participants were selected from the NESARC-III sample to investigate the factor structure, clinical validity, and psychiatric correlates	NESARC • Specific substance use: Marijuana • Sample size: *n* = 3732 • Response rate: *n* = 81% • Survey duration: 2001–2002	Construct validity (convergent validity) • Comparison measure: AUDASIS-IV • Statistical analysis: Factor analysis Criterion validity (predictive validity) • Comparison measure: DSM-IV marijuana dependence criteria • Statistical analysis: Binomial regression	Both marijuana withdrawal symptoms were associated with significant distress/impairment, substance use to relieve/avoid marijuana withdrawal symptoms, and quantity of marijuana use. Panic and personality disorders were associated with anxiety symptoms in both frequent marijuana users and marijuana-only users	4
Hser, Shen [30]	• The overall mean age was 32.6 years (*SD* = 7.6) • Two-thirds (66.0%) of the DATOS sample were male • Almost half of the patients (46.6%) were African American, 38.3% were white, 12.5% were Hispanic, and the remainder (2.7%) were either Asian or others	Non-population-based study conducted in the inpatient and outpatient clinical setting to develop a lifetime severity index for cocaine use disorder and examine its predictive validity of posttreatment outcome using data from the nation Drug Abuse Treatment Outcome Study	LSI-Cocaine • Specific substance use: Cocaine use disorder • Sample size: *n* = 2107 • Response rate: Not reported • Survey duration: 1993–1999	Criterion validity (concurrent validity) • Comparison measure: Not reported • Statistical analysis: Concordance agreement	A higher value of the index, indicating greater severity, predicted a greater likelihood of relapse (the odds ratios were 5.7 for high severity and 4.4 for medium severity, relative to low severity) and shorter time to relapse. Similarly, the polytomous logistic analysis indicated that the index predicted levels of posttreatment cocaine use (odds ratios of daily use were 47.8 for the high severity and 18.8 for medium severity; the corresponding odds ratios of weekly use were 6.75 and 5.10 and for less than weekly use were 3.35 and 3.57)	3
Jackson, Covell [31]	• Mean age (SD) = 37 (7.8) • 71% males and 29% females • 41% were White, and 59% were African American/Black	Non-population-based study conducted in the outpatient clinical setting to clarify the concordance rates of self-report of drug use compared to results of urine screens for drugs of abuse among individuals diagnosed with both a mental health and a substance use disorder	Self-reported drug use • Specific substance use: Not reported • Sample size: *n* = 196 • Response rate: Not reported • Survey duration: 1992–1998	Criterion validity (concurrent validity) • Comparison measure: Urine drug screen • Statistical analysis: Concordance agreement (concordance between self-reported and urine drug screen)	The concordance between self-report and results from urine screens was high. Estimates for the likelihood of use of marijuana and cocaine within the past 30 days were 15% and 32%, respectively, based on urine screens, 25% and 35% based on self-report, and 28% and 43% based on information from both sources combined	5
Joyner, Wright [32]	• Mean age = 30 years • 70% males and 30% females • 22% were White; 78% were African American/Black	Non-population-based study conducted in inpatient clinical setting to examine the ability of the ASI to provide valid and reliable data within a homeless population of drug misusers	ASI • Specific substance use: Not reported • Sample size: *n* = 23 • Response rate: 100% • Survey duration: May–June 1991	Criterion validity (concurrent validity) • Comparison measure: Correlation between ASI section and corresponding composite measure • Statistical analysis: Pearson product-moment correlation coefficients	Only three of the coefficients did not meet this criterion. Years of education and employment income were more highly correlated with composites outside the area of employment. The legal composite rating was more highly correlated with days of cocaine/crack use rather than length of last incarceration. The mean composite score in the study sample was 0.102 for those with no alcohol problem, 0.403 for those with alcohol as a secondary problem, and 0.694 for those identifying alcohol as their primary problem	4
Kellogg, Ho [33]	All drug positive participants: Mean age (SD) = 40.0 (9.8), most participants were male (64%), Caucasian (38%), African American (29%), Hispanic (26%), Other/mixed race (7%), mean education year (SD) = 12.5 (2.5) Normal volunteers: Mean age (SD) = 35.7 (14.1), male participants 49%, Caucasian (56%), African American (16%), Hispanic (14%), Other/mixed race (1%), Asian (13%), mean education year (SD) = 15.7 (3.2)	Non-population-based study conducted in the inpatient and outpatient clinical setting to assess the validity of the DRG scale through multiple analyses	PAI DRG • Specific substance use: Not reported • Sample size: *n* = 370 • Response rate: Not reported • Survey duration: Not reported	Construct validity • Comparison measure: Compared with ASI drug composite scores and ASI drug severity ratings • Statistical analysis: Kruskal–Wallis test Content validity • Comparison measure: Not reported • Statistical analysis: Sensitivity and specificity	The Kruskal–Wallis tests revealed that there were significant correlations among the PAI DRG scale and the ASI scales related to the frequency of use, negative consequences of use, and need and desire for treatment	5
Kupetz, Klagsbrun [34]	Not reported	Non-population-based study conducted in the inpatient and outpatient clinical setting to develop a self-assessment survey instrument for the purpose of detecting abuse of both alcohol and other drugs and to assess validity	SUAS • Specific substance use: Not reported • Sample size: *n* = 5745 • Response rate: 88% • Survey duration: Not reported	Construct validity (convergent validity) • Comparison measure: Medical chart • Statistical analysis: Prevalence, prevalence percent agreement, detection	The SUAS was far superior to patient charts in identifying heavy drug and/or alcohol use, especially when both quantity-frequency and social consequences criteria were considered. Also, the SUAS did at least in simply identifying current heavy use of substances, although the numbers are too small to permit definite conclusions. However, an internal validity, which is the correlation of various parts of the SUAS, was not included in the present study	3
Leonhard, Mulvey [35]	• Mean age (SD) = 34.46 (9.82) • More participants were males (72.2% vs. female 27.1%) • White (47.4%), African American/Black (38.1%), American Indian/Alaska Native (0.7%), Asian (0.5%), Other race (11.3%)	Non-population-based study conducted in a busy online inpatient alcohol and drug abuse clinical setting without tight procedural controls to investigate the internal consistency and validity	ASI • Specific substance use: Not reported • Sample size: *n* = 8984 • Response rate: 90% • Survey duration: Not reported	Construct validity (convergent and discriminant validity) • Comparison measure: Interviewer severity ratings and composite scores • Statistical analysis: Pearson correlation coefficients	Validity analyses showed good promise. Correlation matrices for both composite scores and severity ratings demonstrated good evidence for discriminant validity in measuring the ASI’s seven dimensions, medical severity, employment problems, alcohol and drug use, legal problems, social difficulties, and psychiatric problems	4
McGovern and Morrison [36]*	• Mean age = 36 years, male: 67% and female: 33% • Most participants were White (50%), followed by Black (42%) and other (1%)	Non-population-based study conducted in the inpatient clinical setting to examine criterion-related validity by comparing the CUAD’s concordance rates with psychiatrist-derived diagnoses	CUAD • Specific substance use: Not reported • Sample size: *n* = 129 • Response rate: Not reported • Survey duration: Not reported	Criterion validity (concurrent validity) • Comparison measure: Comparing between CUAD with the MAST and DAST instruments • Statistical analysis: Comparison values in parentheses (%)	The CUAD total severity score is positively associated with all three criterion measures at highly significant levels. The CUAD total severity score is able to significantly discriminate the level of care assignments (*p* < .001). The validity of the CUAD is reported and appear satisfactory	4
	• Mean age = 35.8 years • Most participants are male (*n* = 259, 74%) • Most participants were White (70%), followed by Black (25%) and Hispanic (5%)	Non-population-based study conducted in the inpatient clinical setting to further investigate the validity of the CUAD by comparing two frequently used measures, MAST and DAST	CUAD • Specific substance use: Not reported • Sample size: *n* = 348 • Response rate: Not reported • Survey duration: Not reported	Construct validity • Comparison measure: The comparison was made by contrasting the CUAD derived DSM-III-R substance use disorder diagnoses with the chart diagnosis determined by the unit psychiatrists • Statistical analysis: Pearson correlation coefficients Criterion validity (concurrent validity and predictive validity) • Comparison measure: Explored by testing the capacity of the CUAD total severity score to distinguish among patients assigned to three levels of substance abuse treatment: inpatient, partial hospitalization, and outpatient • Statistical analysis: Relating the instrument concurrently with subjects scored on the MAST and DAST and predictively with the assignment of patients to varying levels of care		4
McNeely, Cleland [37]	• Age ranges from 21 to 65, mean age (SD) = 46 (12), median age = 48 • More participants were female (*n* = 236, 51.4%), followed by male (*n* = 211, 48.1%) • More participants were Black/African American (*n* = 238, 51.0%), followed by Hispanic (*n* = 93, 20.2%), White (*n* = 88, 19.1%), Other (*n* = 38, 8.2%) • Most of participants had education on some college or trade school (*n* = 116, 25.3%)	Non-population-based study conducted in the inpatient clinical setting to test the sensitivity and specificity of SISQs for alcohol and other drug use, as well as their feasibility, for self-administration	SISQs • Specific substance use: Not reported • Sample size: *n* = 459 • Response rate: Not reported • Survey duration: June–July 2012 (site A), November 2013–June 2013 (site B)	Construct validity (discriminant validity) • Comparison measure: Not reported • Statistical analysis: Sensitivity, specificity, positive and negative diagnostic likelihood ratios, ROC curves	The SISQ drug had sensitivity of 71.3% (95% *CI* 62.4–79.1) and specificity of 94.3% (95% *CI* 91.3–96.6), *AUC* = 0.83 (95% *CI* 0.79–0.87), for detecting unhealthy drug use, and sensitivity of 85.1 (95% *CI* 75.0–92.3) and specificity of 88.6% (95% *CI* 85.0–91.6), *AUC* = 0.87 (95% *CI* 0.83–0.91), for drug use disorder	4
McNeely, Strauss [38]	• Age ranges from 21 to 65, mean age (SD) = 46 (11.8), median age = 49 • Males and females were equally distributed (*n* = 292, 49.8%) • More participants were Black/African American (*n* = 293, 50.2%), followed by Hispanic (*n* = 127, 21.7%), White (*n* = 109, 18.7%), Other (*n* = 51, 8.7%) • Most of participants had HS grad or GED degree (*n* = 199, 34.0%)	Non-population-based study conducted in the adult inpatient clinical setting to evaluate the validity and test–retest reliability of the SUBS	SUBS • Specific substance use: Not reported • Sample size: *n* = 586 • Response rate: Not reported • Survey duration: April 2011–April 2012 (site A); June–July 2012 (site B)	Criterion validity (concurrent validity) • Comparison measure: Compared with participants with saliva drug testing, the reference standard measures were used in combination to identify unhealthy use and substance use disorder, for each of the four substance classes in the SUBS • Statistical analysis: Pearson correlation coefficients	For unhealthy use of illicit or prescription drugs, sensitivity was 82.5% (95% *CI* 75.7 to 88.0) and specificity 91.1% (95% *CI* 87.9 to 93.6). Analyses of area under the receiver operating curve (AUC) indicated good discrimination (*AUC* 0.74–0.97) for all substance classes	4
Miele, Carpenter [39]	• Mean age (SD) = 35.6 (8.7) • Most participants were males (62%), non-Hispanic origin White (61%), African American (26%), and 13% Hispanic • 23% had not completed high school	Non-population-based study conducted in the inpatient clinical setting to investigate the test–retest reliability, internal consistency, diagnostic concordance, and concurrent validity of the SDSS’s ICD-10 dependence and harmful use scales	SDSS • Specific substance use: Marijuana use • Sample size: *n* = 180 • Response rate: Not reported • Survey duration: Not reported	Criterion validity (concurrent validity) • Comparison measure: Compared with ICD-10 dependence diagnosis • Statistical analysis: Bivariate correlations between CUDIT-R composite scores and measures of the frequency of marijuana use per week and marijuana-related consequences	The ICD-10 dependence and harmful use severity scales were significantly associated with GAS scores for heroin. For cannabis, the 3 ICD-10 dependence scales and the frequency of harmful use symptoms scale were significantly associated with the number of days of cannabis use	4
O'Hare, Cutler [40]	• Mean age was 44.5 years old • More participants were females (54.2%) than males (45.8%) • The majority of participants were White (90.7%)	Non-population-based study conducted in the outpatient clinical setting to examine preliminary validity and reliability of the SSPI and test the concurrent validity of the SRSA, QFI, and one-item frequency of marijuana use index by examining their intercorrelations	SSPI • Specific substance use: Not reported • Specific mental health: Multidimensional psychosocial distress scale • Sample size: *n* = 227 • Response rate = 76.4% • Survey duration: Spring–summer of 1999	Content validity (factorial validity) • Comparison measure: Not reported • Statistical analysis: Principal components analysis with varimax rotation Construct validity (specific type not reported) • Comparison measure: Self-related SRSA, a QFI, and a one-item index measuring the frequency of marijuana use • Statistical analysis: Coefficient correlations (Spearman’s rho)-correlations among substance abuse indices	Correlations among the one-item self-reported substance abuse index (SRSA), QFI (average drinks per week), and frequency of marijuana use were moderate and significant. Correlations among SSPI subscales and these three substance abuse indices tended to be insignificant overall, or if significant, very low	4
			SRSA, QFI, and one-item index measuring the frequency of marijuana use • Specific substance use: Substance abuse • Sample size: *n* = 227 • Response rate = 76.4% • Survey duration: Spring–summer, 1999	Construct validity (specific type not reported) • Comparison measure: Not reported • Statistical analysis: Coefficient correlations (Spearman’s rho): correlations with brief SSPI subscales		
Peters, Greenbaum [41]	• Mean age (SD) = 32.6 (10.2) years • All the participants were males • White (32.7%), African American (44.9%), and Other race (22.5%)	Non-population-based study conducted among a sample of 400 male inmates in the Holiday Transfer Facility to evaluate the effectiveness of eight different substance abuse screening instruments included overall accuracy, PPV, and sensitivity	ASI, DAST, SASSI-2, SSI, and TCUDS • Specific substance use: Not reported • Sample size: *n* = 400 • Response rate = 75% • Survey duration: Feb–April 1996	Criterion validity (concurrent validity) • Comparison measure: SCID • Statistical analysis: Sensitivity, specificity, PPV, NPV	The TCUDS, SASSI-2, and SSI were examined with respect to their utility to identify either alcohol or drug disorders, while other screening instruments (including independent alcohol and drug screens from the ASI) were examined with respect to alcohol or drug disorders. The positive predictive value in detecting either alcohol or drug dependence was highest for the TCUDS, followed by the ADS/ASI drug, the SSI, and the SASSI-2. Sensitivity of the multipurpose instruments in detecting either alcohol or drug dependence was highest for the SSI, followed by the ADS/ASI drug, the SASSI-2, and the TCUDS	5
Ramsay, Abedi [42]	• Mean age (SD) = 23.5 (4.6) years • More participants were males (70%) • The majority of participants were African American (91.7%). Other race (5%)	Non-population-based study conducted in the inpatient clinical setting to describe the development and initial validation of two new, multidimensional measures of substance use, LSUR and LSUR-12, and to provide structured visual tools to aid recall and enhance the validity of the data	LSUR • Specific substance use: Not reported • Sample size: *n* = 60 • Response rate: Not reported • Survey duration: Not reported	Construct validity (hypothesis testing validity) • Comparison measure: Comparing LSUR and LSUR-12 scores for each substance use and a set of key scores from the LSUR and LSUR-13 • Statistical analysis: Spearman correlations, independent samples Student’s *t*-tests, and Mann–Whitney *U*-tests	Lifetime and past 12-week doses measured by the LSUR and LSUR-12 were highly correlated with the HONC and FTND scores (ranging from *ρ* = 0.796 to *ρ* = 0.852). The number of years of education completed was negatively correlated with the lifetime nicotine dose, as predicted (*ρ* = − 0.351). The LSUR can be used to obtain total alcohol consumption for the 5 years preceding assessment, which had an even stronger correlation with the RETROSUB (*ρ* = 0.768, *p* < .001, *n* = 43). The LSUR can also estimate the number of days of cannabis use in the 5 years preceding the assessment, and for this, a stronger correlation was noted with the number of days of drugs use measured by the RETROSUB (*ρ* = 0.726, *p* < .001, *n* = 41)	4
			LSUR-12 • Specific substance use: Not reported • Sample size: *n* = 60 • Response rate: Not reported • Survey duration: Not reported	Construct validity (hypothesis testing validity) • Comparison measure: Comparing LSUR and LSUR-12 scores for each substance use and a set of key scores from the LSUR and LSUR-13 • Statistical analysis: Spearman correlations, independent samples Student’s *t*-tests, and Mann–Whitney *U*-tests		
Rosenberg, Drake [43]	• Mean age (SD) = 38.03 (8.82) years • More participants were females (52.2% vs. 48.8% for males) • The majority of participants were White (98.4%). Other race (1.6%)	Non-population-based study conducted in the outpatient clinical setting to test the validity of DALI compared with clinician diagnosis and other screens for substance use disorders	DALI • Specific substance use: Not reported • Sample size: *n* = 247 • Response rate: Not reported • Survey duration: 1994–1996	Criterion validity (concurrent validity) • Comparison measure: Clinician diagnosis • Statistical analysis: ROC analysis	ROC curves showed that the DALI functioned significantly better than traditional instruments for both alcohol and drug use disorders	5
Salyers, Bosworth [44]	• Mean age (SD) = 42.3 (10.1) • More participants were females (64.8% vs. 35.2% for males) • White (47.1%), African American (44.6%), and Other race (5.5%)	Non-population-based study conducted in the inpatient and outpatient setting with several mental illness to examine the internal factor structure of the SF-12, test–retest reliability, and convergent and divergent validity by comparing SF-12 scores to other indexes of physical and mental health	SF-12 • Specific substance use: Not reported • Sample size: *n* = 801 • Response rate: Not reported • Survey duration: June 1997–December 1998	Construct validity (specific type of validity not reported) • Comparison measure: Not reported in detail, only specify making comparison between SF-12 to other indexes of physical and mental health • Statistical analysis: Coefficient correlations	Each of the physical health indexes were significantly related to PCS as well as to MCS. However, psychiatric hospitalization and substance use disorder were associated with MCS but not with PCS. Chronic health problems and doctor visits for physical health were more strongly related to PCS than to MCS. However, the correlations for physical health hospitalizations did not differ significantly between PCS and MCS. Mental health indexes of self-reported overall mental health, psychiatric hospitalization, and substance use disorder were more strongly related to MCS than to PCS	1
Schultz, Bassett [45]	• Mean age (SD) = 20.03 (1.51) • Most of participants were females (70.1%) and White (87.8%)	Sub-population-based study conducted in a large, public southeastern university to evaluate the internal consistency, concurrent and discriminant validity, and item performance of CUDIT-R among college students who reported recent marijuana use	CUDIT-R • Specific substance use: Marijuana use • Sample size: *n* = 229 • Response rate: Not reported • Survey duration: Not reported	Construct validity (discriminant validity) • Comparison measure: DSM-5 diagnostic severity levels • Statistical analysis: Sensitivity, specificity, ROC curve Criterion validity (concurrent validity) • Comparison measure: Compared to scores and measures of the frequency of marijuana use and marijuana-related consequences • Statistical analysis: Bivariate correlations between CUDIT-R composite scores and measures of the frequency of marijuana use per week and marijuana-related consequences	The CUDIT-R showed good internal consistency and concurrent validity with cannabis-related outcome measures including frequency of use, cannabis-related consequences, and total DSM-5 criteria endorsed. The CUDIT-R also showed evidence of discriminant validity across DSM-5 severity classifications, achieved high levels of sensitivity (0.929) and specificity (0.704), and excellent area under the receiver operating characteristics curve when using a cutoff score of six. All items displayed high levels of discrimination and varied in terms of difficulty and information provided	5
Schwartz, McNeely [46]	• Mean age (SD) = 46.0 (14.7) • More participants were females (*n* = 1124, 56.2%) compared to males (*n* = 874, 43.7%) • More participants were African-American (*n* = 1112, 55.6%), followed by White (*n* = 667, 33.4%), Other race (*n* = 113, 5.7%), multiracial (*n* = 66, 3.3%), Asian (*n* = 35, 1.8%)	Non-population-based study conducted in the inpatient clinical setting to examine the performance of a TAPS tool compared to the WHO ASSIST by conducting concurrent validity	TAPS • Specific substance use: Not reported • Sample size: *n* = 2000 • Response rate: Not reported • Survey duration: August 2014–April 2015	Criterion validity (concurrent validity) • Comparison measure: Comparison to the full WHO ASSIST as part of a large, multi-site study in eastern US primary care patients • Statistical analysis: Sensitivity, specificity, and PPV and NPV were calculated. ROC curves were computed, and the AUC was examined	For illicit drugs, sensitivities were ≥ 0.82 and specificities ≥ 0.92. The TAPS (at a cutoff of 1) had good sensitivity and specificity for moderate-risk tobacco use (0.83 and 0.97) and alcohol (0.83 and 0.74). Among illicit drugs, sensitivity was acceptable for moderate risk of marijuana (0.71), while it was low for all other illicit drugs and nonmedical use of prescription medications Specificities were 0.97 or higher for all illicit drugs and prescription medications	5
Smith, Bennett [47]	• Age ranges from 18–25, mean age (SD) = 21.0 (2.39), 35% (*N* = 3396) were females • More participants were Whites (*n* = 5196, 53%), followed by African American (*n* = 1563, 16%), Latino (*n* = 1907, 19%), and Other (*n* = 1147, 12%)	Non-population-based study conducted in the outpatient clinical setting to examine the sensitivity and specificity of the SDScrY for three past year criterion variables, AOD, AUD, or DUD, in predicting emerging adults (18–25) substance use disorders	GAIN Short SDScrY • Specific substance use: Not reported • Sample size: *n* = 804 • Response rate: Not reported • Survey duration: Not reported	Criterion validity (predictive validity) • Comparison measure: Compared to the past year AOD, AUD, and DUD • Statistical analysis: Sensitivity, specificity, and the ROC curve	Analyses revealed a high correlation between the SDScrY screener and its longer parent scale (*r* = 0.95, *p* < 0.001). Sensitivity (83%) and specificity (95%) were highest at a cutoff score of two (*AUC* = 94%) on the SDScrY for any past year substance use disorder. Sensitivity (85%) was also high at a cutoff score of two on the SDScrY for any past year alcohol disorder	4
Smith, Cheng [48]	• Age ranges from 21–86, median age = 49, 54% were females • The majority of participants (63%) identified themselves as Black or African American	Non-population-based study conducted in the inpatient clinical setting to test the validity of a single question for unhealthy alcohol use to detect other drug use	SIP-DU • Specific substance use: Not reported • Sample size: *n* = 286 • Response rate: 73% • Survey duration: October 2006–June 2007	Content validity • Comparison measure: Not reported • Statistical analysis: Sensitivity, specificity, likelihood ratios, and AUC curve	The single-question screen at a cutoff of one or more times (the value considered a positive test for alcohol screening) was 67.6% sensitive (95% confidence interval (CI), 50.2–82.0%) and 64.7% specific (95% *CI*, 58.4–70.6%) for the detection of a current drug use disorder. It appeared slightly less sensitive (62.6%; 95% *CI*, 52.3–72.2%) and more specific (72.7%; 95% *CI*, 65.8–79.0%) for the detection of current drug use (although CIs overlapped). If oral fluid test results were considered, the sensitivity for detecting current drug use was lower (58.8%; 95% *CI*, 47.6–69.4%) and the specificity higher (80.3%; 95% *CI*, 72.5–86.7%)	2
Smith, Schmidt [49]	Total study population • Age: Age range from 21 to 86, mean (SD) = 49 (12.3) • Sex: *n* = 115 (54.2%) were women • Race: American Indian/Alaskan native: *n* = 8 (2.8%), Asian: *n* = 7 (2.4%), Black: *n* = 179 (62.6%), Native Hawaiian: *n* = 3 (1.1%), White: *n* = 49 (17.1%), unknown: *n* = 40 (14.0%), Hispanic: *n* = 46 (16.1%) • Education: Some high school: *n* = 81 (28.3%), high school graduate: *n* = 107 (37.4%), some college: *n* = 59 (20.6%), college graduate: *n* = 28 (9.8%), postgraduate education: *n* = 11 (3.9%) Consent to oral fluid testing • Age: Age ranges from 21 to 86, mean (SD) = 49.3 (12.8) • Sex: *n* = 135 (56.2%) were women • Race: American Indian/Alaskan native: *n* = 5 (2.1%), Asian: *n* = 5 (2.1%); Black: *n* = 153 (63.8%), Native Hawaiian: *n* = 2 (0.8%), White: *n* = 42 (17.4%), unknown: *n* = 33 (13.8%), Hispanic: *n* = 38 (15.8%) • Education: Some high school: *n* = 68 (28.4%), high school graduate: *n* = 86 (35.8%), some college: *n* = 50 (20.8%), college graduate: *n* = 26 (10.8%), postgraduate education: *n* = 10 (4.2%)	Non-population-based study conducted in the inpatient clinical setting to validate a single-question screening test for drug use and drug use disorders	SIP-DU • Specific substance use: Not reported • Sample size: *n* = 286 • Response rate: 73% • Survey duration: October 2006–June 2007	Content validity • Comparison measure: DAST-10 • Statistical analysis: Sensitivity, specificity, likelihood ratios, and AUC curve	The single screening question was 100% sensitive (95% confidence interval [CI], 90.6–100%) and 73.5% specific (95% *CI*, 67.7–78.6%) for the detection of a drug use disorder. It was less sensitive for the detection of self-reported current drug use (92.9%; 95% *CI*, 86.1–96.5%) and drug use detected by oral fluid testing or self-report (81.8%; 95% *CI*, 72.5–88.5%). Test characteristics were similar to those of the DAST-10 and were affected very little by participant demographic characteristics	5
Tarter and Kirisci [50]	Group I: Alcohol and drug abusers • Age: Mean (SD) = 40.9 (5.7) • Sex: Male: *n* = 56 (47.1%), female: *n* = 63 (52.9%) • Race: Euro-American: *n* = 104 (87.4%), African American: *n* = 14 (11.8%), Other: *n* = 1 (0.8%) Group II: Normal controls • Age: Mean (SD) = 41.18 (4.79) • Sex: Male: *n* = 59 (49.6%), female: *n* = 60 (50.4%) • Race: Euro-American: *n* = 108 (90.8%), African American: *n* = 11 (9.2%), Other: not reported	Non-population-based study conducted in the inpatient clinical setting to evaluate the structure and psychometric properties of the adult version of the DUSI, to determine the capacity of the DUSI to discriminate substance abusers from non-substance abusing individuals, and to test the sensitivity of the DUSI for detecting individual who qualify for a DSM-III-R diagnosis of abuse or dependence	DUSI • Specific substance use: Not reported • Sample size: *n* = 238 (Group I); *n* = 299 (Group II) • Response rate: 73% • Survey duration: Not reported	Construct validity • Comparison measure: Compared to other measures, such as Multidimensional Personality Questionnaire, Family Assessment Measure, and General Health Questionnaire • Statistical analysis: The scales of the DUSI were correlated with other measures to examine the construct validity of the scales	Each of the 10 DUSI domains is unidimensional. Inter-item, split half, and internal reliability ranged from good to excellent. A score of 4 or higher on the substance use domain correctly classified 80% of the substance abusers, whereas a score of 3 or less accurately detected 100% of the normal control subjects. These results demonstrate that the DUSI is a practical and psychometrically sound screening instrument	5
Tiet, Leyva [51]	• Mean age (SD) = 62.2 (12.6) years • The majority of participants were males (95.25%) • Whites (53.7%) and other race (45.05%)	Non-population-based study conducted in the outpatient clinical setting to create and validate a two-item screen for drug use from the ASSIST (excluding tobacco and alcohol) and to improve the efficiency of screening of drug misuse in primary care	ASSIT drug • Specific substance use: Not reported • Sample size: *n* = 1283 • Response rate: Not reported • Survey duration: Feb 2012–April 2014	Criterion validity (concurrent validity) • Comparison measure: MINI and the inventory of drug use consequences • Statistical analysis: Sensitivity, specificity	Based on the development sample, the ASSIST-Drug was 94.1% sensitivity and 98.6% specific for drug use disorders. Based on the validation sample, it was 95.4% sensitive and 87.8% specific	4
Tiet, Leyva [52]	• Mean age (SD) = 62.2 (12.6) years • The majority of participants were males (95.25%) • Whites (53.7%) and Other race (45.05%)	Non-population-based study conducted in the outpatient clinical setting to examine the concurrent diagnostic accuracy of the SoDU in helping to detect marijuana use disorder	SoDU • Specific substance use: Marijuana use • Sample size: *n* = 1283 • Response rate: Not reported • Survey duration: Feb 2012–April 2014	Criterion validity (concurrent validity) • Comparison measure: MINI • Statistical analysis: ROC analysis, sensitivity, specificity	The SoDU was 100% sensitive and 87.5% specific. When tested in subgroups of patients varying in age, gender, race/ethnicity, marital status, education level, and PTSD status, the SoDU maintained 100% sensitivity in all subgroups; specificity ranged from 76.26 to 94.34%	4
Tiet, Leyva [53]	Total • Mean age (SD) = 62.2 (12.6) years • The majority of participants were males (95.25%) • Whites (53.7%) and Other race (45.05%) • *N* = 969 (75.5%) completed higher than high school education Validation • Mean age (SD) = 62.63 (13.01) • The majority of participants were males (95.3%) • Whites (57.5%) • *N* = 485 (75.8%) completed higher than high school education	Non-population-based study conducted in the outpatient clinical setting to develop and validate the SoDU for diagnostic accuracy by conducting item performance analyses and to examine the performance	SoDU • Specific substance use: Not reported • Sample size: Total: *n* = 1283, validation: *n* = 640 • Response rate: Not reported • Survey duration: Feb 2012–April 2014	Criterion validity (concurrent validity) • Comparison measure: The Mini-International Diagnostic Interview was used as the criterion for DUDs, and the Inventory of Drug Use Consequences was used as the criterion for NCDU • Statistical analysis: ROC analysis, sensitivity, specificity	The screening instrument was 100% sensitive and 93.73% specific for DUDs; when replicated in the second half of the sample, it was 92.31% sensitive and 92.87% specific. The screening instrument was 93.18% sensitive and 96.03% specific for NCDU; when replicated in the second half of the sample, it was 83.17% sensitive and 96.85% specific	4
Tiet, Leyva [5]	• Mean age (SD) = 62.2 (12.6) years • The majority of participants were males (95.25%) • Whites (53.7%) and Other race (45.05%)	Non-population-based study conducted in the outpatient clinical setting to validate brief DAST and to test if the briefer version of the DAST is practical for routine use in primary settings	DAST • Specific substance use: Not reported • Sample size: *n* = 1283 • Response rate: Not reported • Survey duration: Feb 2012–April 2014	Criterion validity (concurrent validity) • Comparison measure: Two criterion measures were used: MINI for drug use disorders and the InDUC for negative consequences of drug use, which includes individuals who may or may not meet criteria for a drug use disorder • Statistical analysis: ROC analysis, sensitivity, specificity	The DAST-2 was 97% sensitive and 91% specific for DUDs in the development sample and 95% sensitive and 89% specific in the validation sample. It was highly sensitive and specific for DUD and negative consequences for drug use in subgroups of patients	4
Tiet, Schutte [54]	• Mean age (SD) = 50.59 (9) years • The majority of participants were males (86.4%) • White (50%), African American/Black (31.8%), and Other race (9.9%)	Non-population-based study conducted in the outpatient clinical setting to conduct AUC, sensitivity, specificity, efficiency, PPV, and NPV of the PCL, PC-PTSD, and five abbreviated versions of the PCL in detecting PTSD for patients seeking treatment in substance use disorder specialty treatment	PTSD PCL-C, PTSD PCL-Bliese-4, PTSD-LS-2, PTSD PCL-LS-3, PTSD-PCL-LS-4, PTSD-PCL-LS-6, PC-PTSD • Specific substance use: Not reported • Specific mental health: PTSD • Sample size: *n* = 242 • Response rate: Not reported • Survey duration: August 2003–December 2004	Criterion validity (concurrent validity) • Comparison measure: C-DIS-IV • Statistical analysis: AUC, cut-point sensitivity, specificity, PPV, NPV, efficient, test + %	Based on the C-DIS-IV, prevalence of PTSD was found to be 36.7 and 52.9% in the SUD and MH samples, respectively. The PCL, PC-PTSD, and five abbreviated versions of the PCL were found to have adequate psychometric properties for screening patients in SUD (AUC ranged from 0.80 to 0.86) and MH (AUC ranged from 0.77 to 0.80) outpatient treatment settings	4
Westermeyer, Crosby [55]	• Mean age (SD) = 30.3 (10.7) • Males (95.25%) and female (43.1%)	Non-population-based study conducted in the outpatient clinical setting to conduct concurrent validity of the M-SAPS compared with three factors: psychiatric-behavioral problems, social-interpersonal problems, and addiction-dependence symptoms	M-SAPS • Specific substance use: Not reported • Sample size: *n* = 642 • Response rate: Not reported • Survey duration: Not reported	Construct validity (convergent validity) • Comparison measure: Not reported • Statistical analysis: Factor analysis Criterion validity (concurrent validity) • Comparison measure: Psychiatric rating, social problem rating scales, assistive use scales • Statistical analysis: Pearson correlation coefficients	All six psychiatric rating scales were most strongly correlated with psychiatric-behavior problems. Additionally, all six comparisons with two social problem measures vs MMADST and Axis V coping scales across the three M-SAPS factors were highly significant. More severe psychosocial stressors in the last year and more substance dependence were associated with higher addictive use symptoms scores	5
Wickersham, Azar [56]	• Mean age (SD) = 47.2 (8.3) • The majority of participants were males (70.1%) • White (19.6%), African American/Black (54.6%), and Other race (25.8%)	Non-population-based study and participants were recruited from a novel jail-release program to conduct the initial validation of the RODS among a sample of 97 newly incarcerated, HIV-positive individuals by comparing the MINI as the primary measure of opioid dependence	RODS • Specific substance use: opioid use • Sample size: *n* = 97 • Response rate: Not reported • Survey duration: 2009–2011	Criterion validity (concurrent validity) • Comparison measure: MINI • Statistical analysis: Concordance analysis, sensitivity, specificity, PPV, NPV	The RODS showed good-to-strong sensitivity (0.97), specificity (0.76), positive predictive value (0.69), and negative predictive value (0.98), while concordance analysis revealed moderate diagnostic agreement (*κ* = 0.67)	4
Woicik, Stewart [57]*	Mean age (SD) = 20 (3.1) years	Sub-population-based study and participants were undergraduate but did not specify the setting to conduct analysis of the internal structure of two versions of the SURPS and to conduct concurrent, discriminant, and incremental validity compared to other theoretically relevant personality and drug use criterion measures	SURPS • Specific substance use: Opioid use • Sample size: *n* = 195 • Response rate: Not reported • Survey duration: Not reported	Construct validity (convergent and discriminant validity) • Comparison measure: Personality scales of NEO-FFI, MAST, PH scores • Statistical analysis: Pearson correlation coefficients Criterion validity (predictive validity) • Comparison measure: Personality scales • Statistical analysis: Hierarchical regression analyses	The first set included the five subscales of the NEO-FFI and accounted for 5% (*R* = .22, pb.08, *ΔR2* = .05) of the variance in MAST scores and 8% of the variance in Ph scores (*R* = 0.28, pb.01, *ΔR2* = .08)	4
	• Mean age (SD) = 19.3 (3.1) years • More females (55.13%) than males (44.87%)	Non-population-based study and participants were recruited from Stony Brook University to conduct test–retest reliability and validity with respect to measuring personality vulnerability to reinforcement-specific substance use patterns	SURPS • Specific substance use: Opioid use • Sample size: *n* = 390 • Response rate: Not reported • Survey duration: Not reported	Construct validity (convergent and discriminant validity) • Comparison measure: DMQ • Statistical analysis: Pearson correlation coefficients	To test the equivalence of item measurements across samples, we applied a more stringent procedure in which all paths (i.e., factor loadings, variances, and covariances) in the model were constrained to be equal for both samples	4
Zanis, McLellan [58]	• Mean age = 39 • Sex: All participants were males • 91% were African American, 8% Caucasian, and 1% Latino; • Education: Average education was 12.4 years	Non-population-based study conducted in the outpatient clinical setting with a sample of 98 homeless substance users awaiting temporary housing placement and shelter to examine the reliability and validity of the ASI	ASI • Specific substance use: Not reported • Sample size: *n* = 98 • Response rate: Not reported • Survey duration: Not reported	Construct validity (discriminant validity) • Comparison measure: Compared the ASI composite score and severity ratings to other tests, including MAST, RAB alcohol, RAB drug, BDI, and SCL-90 • Statistical analysis: Correlations between ASI subscale and appropriate comparable test Criterion validity (concurrent validity) • Comparison measure: (1) Assessed by comparing a test measure to a known conceptually similar standard measure at the same point in time; in (2) a separate evaluation of the concurrent validity of the ASI drug scale, authors examined data from 25 subjects that had drug metabolites detected in a urine sample obtained during the first interview and compared this result with their self-reported use of drugs during the 30-day assessment period covered by the ASI interview • Statistical analysis: Correlations	Both composite score and severity rating measures were found to be quite independent with low intercorrelations. Three of the seven ASI composite scores were tested for and found to have moderate concurrent validity: alcohol (*r* = 0.31 to 0.36), drug (*r* = 0.46), and psychiatric (*r* = S3 to 0.66). Composite score interitem correlations were 0.70 or greater in each of the domains except for employment (0.50) and family (0.52)	5
Zanis, McLellan [59]	• Mean age (SD) = 62 (6.95) years • The majority of participants were males (74.2%) • White (43.5%), African American/Black (56.5%)	Non-population-based study conducted in the inpatient clinical setting to examine aspects of reliability, validity, and utility of ASI among individuals with severe and persistent mental illness and concurrent substance abuse disorders	ASI • Specific substance use: Not reported • Sample size: *n* = 62 • Response rate: Not reported • Survey duration: Not reported	Criterion validity (concurrent validity) • Comparison measure: Compared with the urine sample at the conclusion of the first ASI interview • Statistical analysis: Not reported in detail	The past 30-day self-reported drug use questions of the ASI have poor concurrent validity when compared with urine screens, which showed that the ASI has limited validity	5

*Abbreviations* (alphabetical): Alcohol or other drug use disorder (*AOD*), Addiction Severity Index (*ASI*), Alcohol, Smoking, and Substance Involvement Screening Test-Drug (ASSIST-Drug), area under the curve (*AUC*), alcohol use disorder (*AUD*), Alcohol Use Disorder and Associated Disabilities Interview Schedule-DSM-IV (*AUDASIS-IV*), Beck Depression Index (*BDI*), Cut down, Annoyed, Guilty, and Eye-Opener Substance Abuse Screening Tool (*CAGE*), the Computerized Diagnostic Interview Schedule for DSM-IV (*C-DIS-IV*), the Cannabis Use Disorders Identification Test-Revised (*CUDIT-R*), Dartmouth Assessment of Lifestyle Instrument (*DALI*), Drug Abuse Screening Test (*DAST*), Drinking Motives Questionnaire (*DMQ*), Diagnostic and Statistical Manual III-Revised (*DSM-III-R*), Diagnostic Statistical Manual-Guided Marijuana Inventory (*DSM-G-MI*), drug use disorder (*DUD*), Global Appraisal of Individual Needs (*GAIN*), Global Appraisal of Individual Needs Quick Version 3 (*GAIN-03*), Healthcare Effectiveness Data and Information Set (*HEDIS*), Inventory of Drug Use Consequences (*InDUC*), Lifetime Severity Index for Cocaine Use Disorder (*LSI*-Cocaine), Michigan Alcoholism Screening Test (*MAST*), Mini International Neuropsychiatric Interview (*MINI*); Short-Form 12-Item Health Survey (*SF-12*), negative consequences of drug use (*NCDU*), neuroticism, extroversion, openness, agreeableness, and conscientiousness (*NEO-FFI*), National Epidemiologic Survey on Alcohol and Related Conditions (*NESARC*), negative predictive value (*NPV*), Parents, Partners, Past, and Pregnancy Plus (4P’s Plus), Personality Assessment Inventory Drug Problem Scale (*PAI DRG*), positive predictive value (*PPV*), PTSD Checklist 2 Item (*PCL-LS-2*), PTSD Checklist 3 Item (*PCL-LS-3*), PTSD Checklist 4 Item (*PCL-LS-4*), PTSD Checklist 6 Item (PCL-LS-6), PTSD Checklist–Civilian version (*PCL-C*), Primary Care-PTSD screen (*PC-PTSD*), Quantity Frequency Index (*QFI*), risk for AIDS behavior (*RAB*), Rapid Opioid Dependence Screen (*RODS*), Substance Abuse Subtle Screening Inventory-3 (*SASSI-3*), Structured Clinical Interview for DSM-IIIR (*SCID*), Symptom Checklist-90 (SCL-90), screen of drug use (*SoDU*); self-rated substance abuse (*SRSA*); Single-Item Screening Questions (*SISQs*), Single Question Used from Short Inventory of Problems-Drug Use (*SIP-DU*), South Shore Problem Inventory-revised (*SSRI*), Substance Abuse Subtle Screening Inventory-2 (*SASSI-2*), Substance Dependence Severity Scale (*SDSS*), Substance Use Brief Screen (*SUBS*), Texas Christian University Drug Screen (*TCUDS*), Substance Use and Abuse Survey (*SUAS*), the Alcohol Use Disorder and Associated Disabilities Interview Schedule (*AUDADIS*), the Chemical Use, Abuse, and Dependence (*CUAD*), the Drug Use Screening Inventory (*DUSI*), the Marijuana Screening Inventory (*MSI-X*), the Simple Screening Instrument (*SSI*), the Simple Screening Instrument for Substance Abuse (*SSI-SA*), the Longitudinal Substance Use Recall Instrument (*LSUR*), the Longitudinal Substance Use Recall Instrument Recall for 12 Weeks instrument (*LSUR-12*), tobacco, alcohol, prescription medication, and other substance use (*TAPS* tool), receiver operating characteristics (*ROC*). *Studies conducted validity testing in two study populations/samples

The included studies were published between 1979 and 2021, with a wide variation in demographic characteristics. Of the 46 studies, seven had over 80% male participants (Han et al., 2017; Peters et al., 2000; Tiet et al., 2016; Tiet et al., 2019; Tiet et al., 2015; Tiet et al., 2017; Zanis et al., 1994). Among these studies, two recruited only male participants (Peters et al., 2000; Zanis et al., 1994). Additionally, there was one study that only recruited female participants [22]. Racial and ethnic differences also existed among these study samples. Six studies had study sample of primarily (70% or more) White participants [41, 58], and three studies had a study population sample of 70% or more Black/African American (AA) participants [32, 42, 58]. Furthermore, 12 studies only recruited White and Black/AA participants [6, 18, 21, 24, 25, 31, 32, 36, 39, 41, 58, 59]. Seven studies (15.2%) did not report information on race/ethnicity characteristics [19, 22, 27, 34, 55, 57].

All 44 studies included in this review reported the final sample size, with a mean of 1427 (median = 449) participants with an overall range of 23–10,167 participants. Only 13 studies reported response rate, and the response rates ranged between 13.4 [18] and 100% [32]. Twenty-six studies reported the survey duration, and it ranged from 1 month [32] to 120 months [20], with mean 28.48 months (median 13 months). Moreover, studies reported the mean age of the participants as < 30 years (*n* = 4), between 30 and 39 years (*n* = 16), and ≥ 40 years (*n* = 18). Another eight studies reported age groups or median age of the study population. Additionally, a majority (*n* = 37) of the studies were conducted in non-population-based clinical settings (e.g., inpatient, outpatient).

### Participant recruitment strategy

The participant recruitment strategies from included studies in this review were shown in Table 2. Of the 46 studies, only 4% (*n* = 2) examined SUD in the general population [20, 28]; the rest (*n* = 44) of the studies were conducted in clinical or other population subgroups. In the first population-based study, 6664 adult Medicaid enrollees were recruited from 1 of 7 Florida regions who took part in the Florida Health Services Survey at least once between 1998 and 2008 [20]. Researchers assessed the internal psychometric properties of the Simple Screening Instrument for Substance Abuse (SSI-SA) but did not compare survey responses with SUD diagnoses in Medicaid clinical records. In the second population-based study, participants were selected from the National Epidemiologic Survey on Alcohol and Related Conditions-III (NESARC-III) sample, which included noninstitutionalized US adult residents (aged 18 years or older) [28]. The authors then selected 777 respondents for the procedural validity study and used a test–retest design to compare concordance of respondents’ answers to the NESARC-III survey questions with a semi-structured interview, the Psychiatric Research Interview for Substance and Mental Disorders, DSM-5 version (PRISM-5), administered by a clinician.

**Table 2:** Participant recruitment strategies

Population based (*n* = 2)	Clinical (*n* = 35)	Non-population based & non-clinical (*n* = 9)
	• Inpatient (*n* = 19)	• Veteran’s administration shelter (*n* = 1)
	• Outpatient (*n* = 11)	• Unspecified drug and alcohol program (*n* = 1)
	• Both inpatient and outpatient (*n* = 5)	• Prison substance use treatment program (*n* = 1)
		• Transfer facility (*n* = 1)
		• Jail release program (*n* = 1)
		• NESARC-III subsample (*n* = 1)
		• University (*n* = 3)

Of the remaining 44 studies not in the general population, over three quarters (*n* = 35) were conducted in the clinical setting, with the majority (*n* = 19) in the inpatient setting [6, 19, 21, 22, 24, 26, 32, 35–39, 42, 46, 48–50, 59]. Eleven studies were conducted in the outpatient clinical setting [5, 23, 31, 40, 43, 47, 51–55], 5 studies were conducted in both the inpatient and outpatient settings (Harris et al., 2015; Hser et al., 1999; Kellogg et al., 2002; Kupetz et al., 1979; Salyers et al., 2000), and 1 study was conducted in a Veterans’ Administration shelter [58]. The remaining studies (*n* = 8) were conducted outside the clinical setting. For example, participants were recruited from an alcohol and drug program [18], prison substance abuse treatment programs [25], Holiday Transfer Facility [41], and a novel jail-release program [56]. Lastly, four studies consisted of sub-population samples within the National Epidemiologic Survey on Alcohol and Related Conditions-III (NESARC-III) [29] and universities using student participants [45, 57].

### Quality of studies

Risk of bias was assessed based upon the methodology used for instrument comparison and the statistical analysis conducted. Although several studies adopted recruitment strategies that limited their study population to specific groups (for example, only recruiting male or white populations), the risk-of-bias assessment employed by the current study did not account for recruitment. As a result, most of the included studies (*n* = 41) had a risk-of-bias score of 4 or higher (Table 1). Two studies had a score of 3 [30, 34], one studies had a score of 2 [29, 48], and two studies had a score of only 1 [27, 44]. Among those studies with low-quality assessment scores, four studies lacked statistical comparisons and reported prevalence estimates only [27, 30, 34, 44]. There were three studies that did not report on validation methodology [27, 44, 48].

### Survey measure

**Fig. 2:**
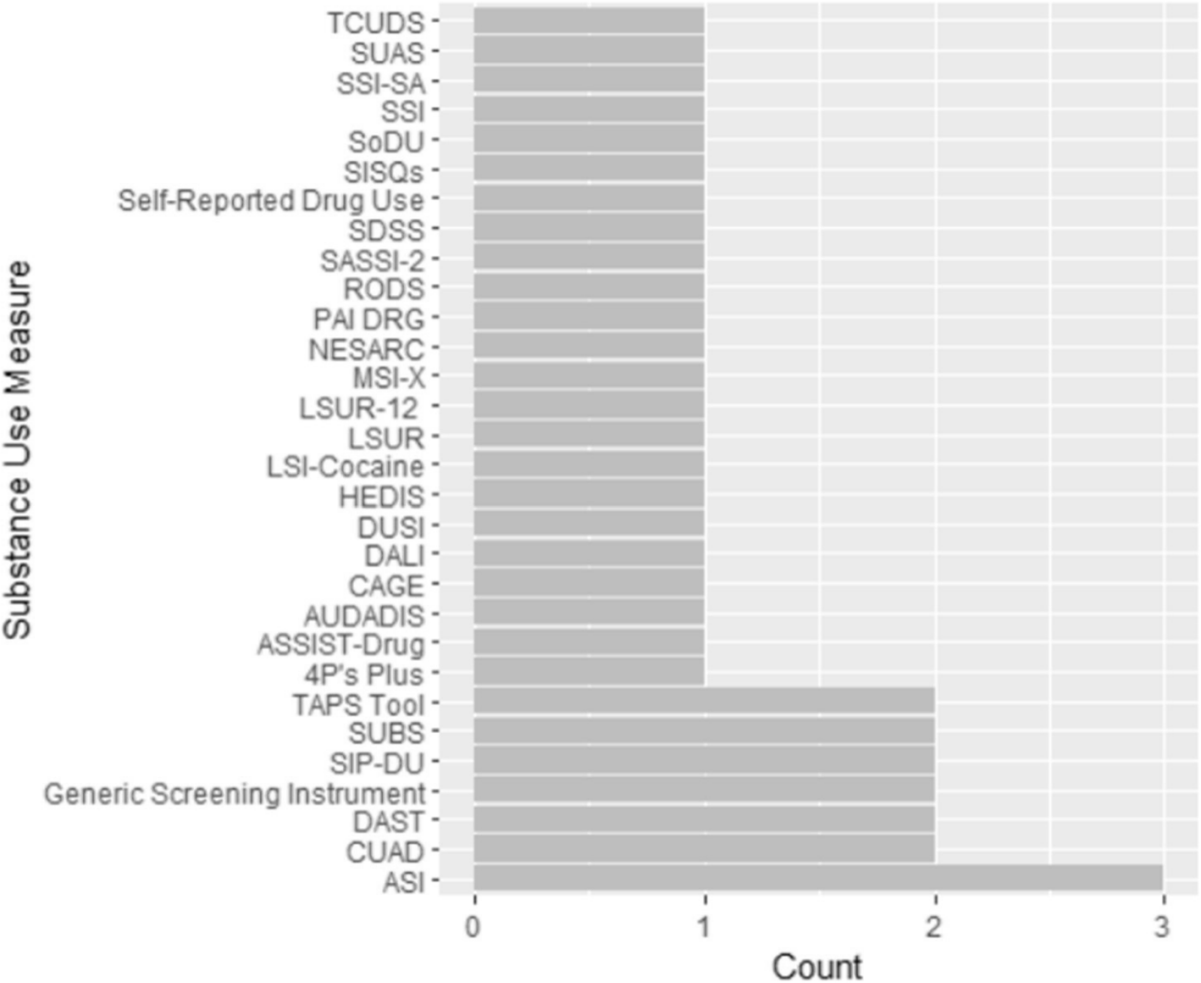
Frequency of survey measures used in included studies. Abbreviations in order: Texas Christian University Drug Screen (TCUDS), Substance Use and Abuse Survey (SUAS), the Simple Screening Instrument for Substance Abuse (SSI-SA), the Simple Screening Instrument (SSI), screen of drug use (SoDU), single-item screening questions (SISQs), Substance Dependence Severity Scale (SDSS), Substance Abuse Subtle Screening Inventory-2 (SASSI-2), Rapid Opioid Dependence Screen (RODS), Personality Assessment Inventory Drug Problem Scale (PAI DRG), National Epidemiologic Survey on Alcohol and Related Conditions (NESARC), the Marijuana Screening Inventory (MSI-X), the Longitudinal Substance Use Recall Instrument Recall for 12 Weeks instrument (LSUR-12), the Longitudinal Substance Use Recall Instrument (LSUR), Lifetime Severity Index for Cocaine Use Disorder (LSI-Cocaine), Healthcare Effectiveness Data and Information Set (HEDIS), the Drug Use Screening Inventory (DUSI), Dartmouth Assessment of Lifestyle Instrument (DALI), Cut down, Annoyed, Guilty, and Eye-Opener Substance Abuse Screening Tool (CAGE), the Alcohol Use Disorder and Associated Disabilities Interview Schedule (AUDADIS), Alcohol, Smoking, and Substance Involvement Screening Test-Drug (ASSIST-Drug), Parents, Partners, Past, and Pregnancy Plus (4P’s Plus), tobacco, alcohol, prescription medication, and other substance use (TAPS tool), Substance Use Brief Screen (SUBS), single question used from short inventory of problems-drug use (SIP-DU), Drug Abuse Screening Test (DAST), the Chemical Use, Abuse, and Dependence (CUAD), Addiction Severity Index (ASI)

Among the articles included in this review, 89% (*n* = 41) used measures specifically designed for screening SUDs. For example, seven studies tested the validity of the measure’s ability to screen for a specific SUD, including marijuana use [18, 21, 29, 40, 45], cocaine use [30], and opioid use [56]. Five studies validated measures for both substance use and mental health [23, 25, 40, 44, 54], of which one study used a measure for post-traumatic stress disorder (PTSD) screening [54]. The rest of the included studies did not specify a specific SUD for screening purposes but used a generic term for defining SUD. All measures and their frequency of use in the included studies are depicted in Fig. 2.

The majority of studies validated one single measure, of which five studies validated the Addiction Severity Index (ASI), [32, 35, 41, 58, 59] and one study validated drug use subscales of ASI [26, 37]. Five studies validated multiple survey measures:

1. Duncan et al. validated two survey measures: (a) CJDAT Co-Occurring Disorders Screening Instruments for any Mental Disorder (CODSI-MD) and (b) CJDAT Co-Occurring Disorders Screening Instruments for Severe Mental Disorder (CODSI-SMD) [25].
2. Ramsay et al. also validated two different survey measures: (a) The Lifetime Substance Use Recall Instrument (LSUR) and (b) the Longitudinal Substance Use Recall for 12 Weeks instrument (LSUR-12) [42].
3. Peters et al. and Tiet et al. also validated two measures: (a) The Substance Use Brief Screen (SUBS) and (b) the DAST [5, 41]. O’Hare et al. validated four different survey measures: (a) South Shore Problem Inventory-revised (SSPI), (b) self-rated substance abuse (SRSA), (c) quantity-frequency index for alcohol consumption (QFI), and (d) one-item index measuring the frequency of marijuana use [40].
4. Peters et al. validated five different survey measurements: (a) ASI-drug use subscales, (b) DAST, (c) Substance Abuse Subtle Screening Inventory-2 (SASSI-2), (d) SSI, and (e) Texas Christian University Drug Screen (TCUDS) [41].
5. Tiet et al. conducted validations of seven survey measures: (a) PTSD Checklist–Civilian version (PCL-C), (b) PTSD Checklist 4 Item (PCL-Bliese-4), (c) PTSD Checklist 2 Item (PCL-LS-2), (d) PTSD Checklist 3 Item (PCL-LS-3), (e) PTSD Checklist 4 Item (PCL-LS-4), (f) PTSD Checklist 6 Item (PCL-LS-6), and (g) Primary Care–PTSD screen (PC-PTSD) [54].

Two studies conducted survey measure validation in different study populations. One study conducted a preliminary exploration of the psychometric properties of the Substance Use Risk Profile Scale (SURPS) in 3 different populations: 195 undergraduate drinkers, 390 undergraduate students from Stony Brook University, and 4234 high school students in Canada [57]. In the second study, data were collected from two separate adult clinical samples — seriously mentally ill inpatients and patients presenting for evaluation at a chemical dependence program — to describe the rationale and test validity and reliability of the Chemical Use, Abuse, and Dependence Scale (CUAD) [36].

### Comparison measures for validation

Several different types of measures were used as comparison for the purpose of validation. Higher quality comparison measures included items such as medical records, diagnoses, medical test results, or other SUD severity scales. A total of 10 studies conducted validity testing using at least one of these higher-quality comparison measures. Of these, three studies conducted criterion validity testing by comparing the following: (1) positive and negative 4P’s Plus screens with positive and negative clinical assessment [22], (2) the Alcohol Use Disorder and Associated Disabilities Interview Schedule (AUDADIS) with psychiatrist diagnosis [28], and (3) Dartmouth Assessment of Lifestyle Instrument (DALI) with clinician diagnosis [43]. The remaining two studies conducted validity testing by comparing the following: (1) Substance Use and Abuse Survey (SUAS) with medical chart [34] and (2) CUDIT-R with DSM-T diagnostic severity levels [45]. Another study compared the CUAD-derived DSM-III-R substance use disorder diagnoses with the chart diagnosis determined by the unit psychiatrists for validation [36].

Furthermore, two studies validated their measures by comparing with diagnostic standards: (1) Compared Cut down, Annoyed, Guilty, and Eye-Opener Substance Abuse Screening Tool (CAGE) with SCID-generated drug use disorder diagnoses as the standard [24] and (2) Compared the Cannabis Use Disorders Identification Test Revised (CUDIT-R) with ICD-10 dependence diagnosis [39]. Three studies conducted validity testing by comparing with laboratory test results, including urine test [31, 58, 59] and saliva drug testing [38].

Four studies conducted validity testing by comparing other severity scales: (1) Criterion validity testing by comparing the Marijuana Screening Inventory (MSI-X) with three different severity rating scales and selected variables [18], (2) construct validity testing by comparing Personality Assessment Inventory Drug Problem Scale (PAI DRG) with ASI drug composite scores and severity ratings [33], (3) construct validity testing by comparing ASI with interviewer severity ratings and composite scores [35], and (4) concurrent validity of ASI drug scale and examined 25 participants who had drug metabolites detected in a urine sample obtained during the first interview and compare this result with their self-reported use of drugs during the 30-day assessment period in ASI interview [58].

### Types of validity assessed and statistical analyses conducted

Two-thirds of the studies (*n* = 30) included in this review examined criterion validity, specifically concurrent validity (*n* = 22), predictive validity (*n* = 5), and specification validity (*n* = 1), and unspecified (*n* = 2). Over half (*n* = 24) studies conducted construct validity, specifically, convergent validity (*n* = 10), discriminant validity (*n* = 6), hypothesis testing validity (*n* = 1), predictive validity (*n* = 2), and factorial validity (*n* = 1). Eight articles did not report specific types of construct validity. While three studies conducted content validity, none reported specific type of content validity [33, 37, 48]. Additionally, 11 studies conducted validity testing for multiple measures [18, 19, 29, 33, 36, 39, 40, 45, 55, 57, 58]. Ten studies investigated construct and criterion validity of a single survey measure [18, 19, 29, 36, 39, 45, 55, 57, 58], and one study conducted construct and content validity of a single survey measure [33].

Studies conducted the following statistical analyses for testing validity of survey measures: (1) sensitivity and specificity (*n* = 16), (2) receiver operating characteristics (ROC) curve (*n* = 10), (3) correlation coefficient (*n* = 9), (4) Pearson correlation coefficient (*n* = 8), and (5) positive predicted value (PPV) (*n* = 8). Sensitivity and specificity were the most common statistical method for validation among studies examining construct validity and criterion validity.

Most studies showed strong evidence of validity or had strong significant associations with other measures for comparison. Studies that compared substance use measures with physician diagnoses or medical records showed strong overall validity. For example, Rosenberg et al. conducted ROC analysis for criterion validity and concluded that DALI functioned significantly better than traditional instruments for substance use disorders among psychiatric patients [43]. Compared with DAST-10, Short Inventory of Problems-Drug Use (SIP-DU) showed 100% sensitivity and 73.5% specificity for the detection for a drug use disorder. It was less sensitive at detecting self-reported current drug use (92.9%) and drug use detected by oral fluid testing or self-report (84.7%) [49]. However, studies demonstrated lack of validity for certain measures. For example:

1. Compared to urine screens, the ASI’s questions about drug use in the past 30 days had poor concurrent validity, which suggested that the ASI has limited validity [59].
2. Correlations were not statistically significant among South Shore Problem Inventory-revised (SSPI) subscales and three other substance abuse indices, such as self-related substance abuse (SRSA), quantity-frequency index (QFI) for alcohol consumption, and one-item index measuring the frequency of marijuana use [40].
3. Compared with oral fluid test results, using SIP-DU at a cut-off score (to be considered a positive test for alcohol screening) showed lower sensitivity and higher specificity for detecting current drug use [48].

## Discussion

This systematic review found 46 studies conducted in the US between 1979 and 2021 that tested the validity of substance use/SUD measures. Two studies were population based [20, 28], while the rest were conducted in subpopulations or in clinical settings. Criterion validity and construct validity were the commonly used validation methods, and sensitivity and specificity were the most common statistical analyses for validation. More importantly, this review found that a myriad of survey measures was used to measure substance use/SUD. In addition, diverse methodologies were applied to measure validity, which makes comparability difficult. In general, most studies showed evidence of strong validity.

For example, among those articles included in this review, 46 studies tested the psychometric properties of 43 different substance use screening measures. Of them, 16 tested the validity of psychometric properties by comparing other self-reported survey measures, and one study conducted criterion validity by comparing different racial or ethnic groups of offenders [25]. Fourteen studies conducted concurrent validity by comparing measures with an external independent source or “gold standard,” such as physician/clinician diagnosis, medical records or assessment, severity scales, or urine/saliva drug testing. Frequently, researchers rely on self-reported information on substance use to save time and cost and collect required information on a larger sample size than making comparison with a gold standard, such as a biological test or medical record.

The measures used in these studies varied greatly. The ASI, which was used most frequently in this review, was used in only five studies. Additionally, three articles specifically conducted validity testing for marijuana use. However, each of those studies used many diverse measures, such as MSI-X [18], the CUDIT-R [39, 45], screen of drug use (SoDU) [60], NESARC [60], a two-item brief screen with no instrument name reported [60], and one-item index measuring the frequency of marijuana use [40]. Multiple measures for one specific substance use might increase the likelihood of conflicting results, which can make it difficult to interpret and compare results across different studies. Thus, there is a need to adopt a standardized measure to ensure the results obtained are reliable and to be able to draw general conclusions.

In addition to the diverse measures, even the validation methods employed in the articles varied greatly. Although criterion and construct validity were the most commonly utilized validity measures, the specific type of criterion or construct validity varied among studies. For example, concurrent, predictive, and specification validity were reported as the three different types of criterion validity. Some studies employed multiple validation methods for a single survey measure, while others only used one. Moreover, different types of validity may achieve different objectives, which could explain the differences in statistical analyses of validation. This review also suggested that the statistical analyses used to test the validity of survey measures were diverse, with sensitivity and specificity being the most frequent analysis. Other statistical analyses such as ROC curve and correlation coefficient were also used to validate the survey measures.

Likewise, other differences were observed for demographic characteristics of participants. First, the validation of the substance use and SUD measures was primarily conducted in either inpatient or outpatient clinical settings, and only two studies were population based. Secondly, some studies had small sample sizes, which could significantly reduce the statistical power for finding differences between study groups. Moreover, some studies were occasionally limited to certain age or race/ethnicity groups, which could adversely affect the generalizability of findings. For example, several studies were restricted to White or Black/AA participants [6, 18, 21, 24, 25, 31, 32, 36, 39, 41, 58, 59]. In addition, information on race/ethnicity was missing from a few studies [19, 22, 27, 34, 55, 57]. Those studies might reflect racial disparities in SUD, as well as treatment for SUD. Although SUD is prevalent among all racial groups, the burden of disease is disproportionate among Black people, and treatment of SUD is less available for Black people [61]. Three studies were limited to either males or females only [22, 41, 58]. These studies provide valuable validation in the respective populations and may prove useful in other populations. However, further validation is needed in diverse populations for these measures to be generalizable.

SUD often co-occurs with many other physical and mental health conditions. Previous studies have shown a high co-occurrence and the increased risk of mental health disorders among individuals with SUD, which can be observed in clinical samples [62, 63]. In this review, only five studies validated measures for both substance use and mental health disorders. Results from studies assessing substance use and mental health simultaneously can help inform integrated treatment interventions by connecting individuals with additional service providers who can provide specialized services to treat the physical and emotional elements of mental health and SUD [64]. Additional advantages of assessing co-occurring substance use and mental health include decreased hospitalization, fewer arrests, and increased housing stability [64]. More importantly, assessing co-occurring substance use and mental health disorders in population research can identify the barriers and disparities of treatment access, including race/ethnicity [65] and low treatment utilization among individuals with only substance use or only mental health disorders [66, 67].

Although this review adhered to the PRISMA guidelines, it is not without limitations. It was limited to studies conducted in the US, and studies in other countries were not included. Research shows that significant contextual differences, such as burden of substance use disorders, cultural norms, legal frameworks, healthcare systems, and societal attitudes towards substance use, can vary widely across countries, potentially influencing the reliability and applicability of measures developed and validated in one context when applied to another [1–3]. Our focus on US-based studies aims to ensure that the measures reviewed are relevant and applicable to the US population, providing a more accurate and context-specific assessment of substance use and SUDs.

Although a rigorous search strategy was implemented, our search was limited to library databases. As such, key clinical surveys were used in hospitals or other specialty clinical settings that were not published in peer-reviewed journals and may be missing from our review. Additionally, our objectives were to summarize the validity of measures to assess the prevalence of substance use and SUD in the US estimated in population and sub-population-based surveys. Therefore, we did not specifically review the best clinical practices for survey administration in the clinical setting. Findings highlight the need to evaluate substance use surveys in a population-based setting to identify a valid survey for use across population-based surveys. The consistent use of one survey may provide for more accurate comparisons across populations. However, the main limitation of this review is that the articles included in this review are missing information about demographic characteristics, such as the distribution of race and ethnicity groups in the study population, and only 5 studies in this review reported education level of the participants [33, 37–39, 49, 58]. The variation in the accuracy of self-reported data about substance use depends on education and socioeconomic status [68]. The majority of studies included in this review did not report the response rate or the survey duration. Lastly, our analyses relied only on peer-review studies, and our review did not include internal studies that may have been conducted in large surveys, such as NSDUH.

This study has several strengths. To our knowledge, it is the first systematic review to summarize the validity of substance use/SUD measures used in questionnaires or instruments among US adults. This review has included 43 years of data among nine different literature databases. In addition, it has also included “gray literature” such as theses and Google Scholar, which can make significant contributions to systematic reviews by minimizing publication bias, enabling a more impartial assessment of the evidence, and publicizing null or negative findings [69]. Another strength of the study is that the methodologic quality of validation studies was assessed by an adapted risk-of-bias tool, created especially for this assessment. Lastly, while previous reviews have explored the instruments used to assess substance use and the identification of disorders [7, 8], this review uniquely concentrates on a comprehensive evaluation of the psychometric properties of measures assessing a broader spectrum of substances. This review aimed to distinguish from previous research, highlighting the diversity and specificity of instruments in current use, their applicability in various population and sub-population surveys, and the critical need for standardized, short, and versatile measures.

The findings of this review have several key implications. The study demonstrates that survey questions can be used to assess the prevalence of SUD in specific populations. However, most studies used different measures suggesting there was no consensus on the best measure to use for assessing the prevalence of substance use and SUD. This lack of common measures illustrates the difficulty in assessing SUD in short surveys, especially for specific substances. Similar to a global measure of psychological distress that is used to indicate nonspecific psychological distress [70], a measure is needed for measuring SUD in population-based studies. Only 5 out of 46 studies were conducted in population or sub-population-based settings. Therefore, more research needs to be conducted to validate these measures in population-based settings to confirm their sensitivity and specificity. Additionally, more studies need to validate measures using a “gold standard,” such as an outside reliable measure, because comparing with self-reported substance use can result in misclassification bias. Therefore, this systematic review illustrates a critical need to develop short measures for assessing SUD that do not require lengthy, time-consuming data collection that would be difficult to incorporate into population-based surveys assessing a multitude of health dimensions.

## Conclusion

This systematic review summarized the validity of measures used to assess the prevalence of substance use and SUD in the US estimated in general population surveys and other population-based settings. Among the 46 studies included, this review demonstrated that a myriad of survey measures were used to assess substance use and SUD, and diverse methodologies were used to measure validity. This information suggests a lack of standardized, comparative survey measures in assessing the prevalence of substance use and SUD among US adults. This inconsistency makes it difficult to recommend the best measures to use in US surveys and highlights the need to develop better summary measures. Very few studies in this review were conducted in general population settings, which suggests that more research is needed to validate substance use measures in such settings. Although SUD is prevalent among all racial/ethnicity, age, and gender/sex groups in the US, and studies in this review provided valuable validation in the respective populations, further validation is needed in diverse populations. Thus, future validation research needs to be conducted in population-based settings to adopt a standardized measure for substance use and SUD that can inform interventions aimed to detect and manage problems associated with substance use and SUD and prevent avoidable premature US deaths.

### Supplementary Information

**Additional file 1:** **Supplementary Table 1.** Search term list for each database. Figure 2. Bar Graph of Survey Measures Validated by Included Studies

## Data Availability

Table 1 contains the extracted data, and supplementary file contains the search strategy.

## Abbreviations

SUD: Substance use disorder
US: United States
DSM: *Diagnostic and Statistical Manual of Mental Disorders*
NSDUH: National Survey of Drug Use and Health
NAVIPPRO™: National Addictions Vigilance Intervention and Prevention Program
ASI-MV®: Addiction Severity Index-Multimedia Version®
DAST: Drug Abuse Screening Test
ASSIST: Alcohol, Smoking, and Substance Involvement Screening Test
TAPS: Tobacco, alcohol, prescription medication, and other substance use
PRISMA: Preferred Reporting Items for Systematic reviews and Meta-Analyses
BRFSS: Behavioral Risk Factor Surveillance System
AA: African American
SSI-SA: Simple Screening Instrument for Substance Abuse
NESARC-III: National Epidemiologic Survey on Alcohol and Related Conditions-III
PRISM-5: Psychiatric Research Interview for Substance and Mental Disorders, DSM-5 version
PTSD: Post-traumatic stress disorder
ASI: Addiction Severity Index
CODSI-MD CJDAT: Co-Occurring Disorders Screening Instruments for any Mental Disorder
CODSI-SMD CJDAT: Co-Occurring Disorders Screening Instruments for Severe Mental Disorder
LSUR: The Lifetime Substance Use Recall instrument
LSUR-12: The Longitudinal Substance Use Recall for 12 Weeks instrument
SSPI: South Shore Problem Inventory-revised
SRSA: Self-rated substance abuse
QFI: Quantity-frequency index for alcohol consumption
SASSI-2: Substance Abuse Subtle Screening Inventory-2
TCUDS: Texas Christian University Drug Screen
PCL-C: PTSD Checklist–Civilian version
PCL-Bliese-4: PTSD Checklist 4 Item
PCL-LS-2: PTSD Checklist 2 Item
PCL-LS-3: PTSD Checklist 3 Item
PCL-LS-4: PTSD Checklist 4 Item
PCL-LS-6: PTSD Checklist 6 Item
PC-PTSD: Primary Care-PTSD screen
SURPS: Substance Use Risk Profile Scale
CUAD: Chemical Use, Abuse, and Dependence Scale
AUDADIS: Associated Disabilities Interview Schedule
DALI: Dartmouth Assessment of Lifestyle Instrument
SUAS: Substance Use and Abuse Survey
CAGE: Cut down, Annoyed, Guilty, and Eye-Opener Substance Abuse Screening Tool
CUDIT-R: Cannabis Use Disorders Identification Test-Revised
MSI-X: Marijuana Screening Inventory
PAI DRG: Personality Assessment Inventory Drug Problem Scale
ROC: Receiver operating characteristics
PPV: Positive predicted value
SIPDU: Short Inventory of Problems-Drug Use
SRSA: Self-related substance abuse
QFI: Quantity-frequency index
SoDU: Screen of drug use
