# Supplementary material for: A systematic review of validity of US survey measures for assessing substance use and substance use disorders
Source: Syst Rev. 2024 Jun 27;13:166. doi: 10.1186/s13643-024-02536-x (PMC11210012; doi:10.1186/s13643-024-02536-x)
Supplement: Supplementary file 1 — Additional file 1: Supplementary Table 1. Search term list for each database. Figure 2. Bar Graph of Survey Measures Validated by Included Studies [file 13643_2024_2536_MOESM1_ESM.docx]

**Supplementary Table 1. Search term list for each database**

| Database | Search strings* | Results |
| --- | --- | --- |
| PubMed | 1. ((surveys and questionnaires[MeSH Terms]) OR (survey [Title/Abstract])) OR (questionnaire [Title/Abstract]) 2. (reproducibility of results[MeSH Terms]) OR (valid*[Title/Abstract]) 3. (health surveys[MeSH Terms]) OR (health survey*[Title/Abstract]) OR (health care surveys[MeSH Terms]) OR (health care survey*[Title/Abstract]) OR (healthcare survey*[Title/Abstract]) 4. (Substance-Related Disorders[MeSH TERMS]) OR (substance use[Title/Abstract]) OR (mental health[Title/Abstract]) OR (mental health[MeSH Terms])     #1 AND #2 AND #3 AND #4 | 4051 |
| Scopus | (AB survey OR TI survey OR AB questionnaire OR TI questionnaire) AND (AB “health survey*” OR TI “health survey*” OR AB “health care survey*” OR TI “health care survey*” OR AB “healthcare survey” OR TI “healthcare survey”) AND (AB valid* OR TI valid*) AND (AB “substance use” OR TI “substance use” OR AB “mental health” OR TI “mental health”) | 661 |
| CINAHL | 1. MH surveys OR MH questionnaires OR AB surveys OR AB questionnaire OR TI survey OR TI questionnaire 2. MH “reproducibility of results” OR AB valid* OR TI valid* 3. AB “health survey*” OR TI “health survey*” OR AB “health care surveys” OR TI “health care surveys” OR AB “healthcare survey*” OR TI “healthcare survey” 4. MH “substance use disorders” OR AB “substance use” OR TI” substance use” OR MH “mental health” OR AB “mental health” OR TI “mental health”     #1 AND #2 AND #3 AND #4 | 293 |
| PsycINFO | 1. MA ( surveys and questionnaires ) OR AB survey OR AB questionnaire 2. MA reproducibility of results OR AB valid* 3. MA “health surveys” OR AB “health survey*” OR MA “health care surveys” OR AB “health care survey*” OR “healthcare survey*” 4. MA “substance-related disorders” OR AB “substance use” OR MA “mental health” OR AB “mental health”     #1 AND #2 AND #3 AND #4 | 569 |
| Academic Search Complete | (AB survey OR TI survey OR AB questionnaire OR TI questionnaire) AND (AB “health survey*” OR TI “health survey*” OR AB “health care survey*” OR TI “health care survey*” OR AB “healthcare survey” OR TI “healthcare survey”) AND (AB valid* OR TI valid*) AND (AB “substance use” OR TI “substance use” OR AB “mental health” OR TI “mental health”) | 357 |
| Web of Science | (AB survey OR TI survey OR AB questionnaire OR TI questionnaire) AND (AB “health survey*” OR TI “health survey*” OR AB “health care survey*” OR TI “health care survey*” OR AB “healthcare survey” OR TI “healthcare survey”) AND (AB valid* OR TI valid*) AND (AB “substance use” OR TI “substance use” OR AB “mental health” OR TI “mental health”) | 583 |
| ProQuest Theses and Dissertation Global | (AB survey OR TI survey OR AB questionnaire OR TI questionnaire) AND (AB “health survey*” OR TI “health survey*” OR AB “health care survey*” OR TI “health care survey*” OR AB “healthcare survey” OR TI “healthcare survey”) AND (AB valid* OR TI valid*) AND (AB “substance use” OR TI “substance use” OR AB “mental health” OR TI “mental health”) | 36 |
| Google Scholar | “Reliability and validity substance use” (n=first 200)  “Reliability and validity mental health” (n=first 200) | 400 |

*Abbreviations: AB=abstract, MH=CINAHL Subject Headings for both major and minor headings, TI=title, MA=MeSH Subject Headings


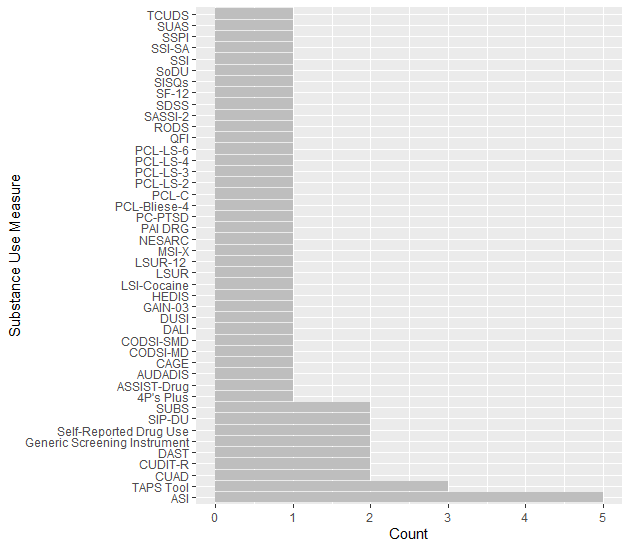


**Figure 2. Bar Graph of Survey Measures Validated by Included Studies**

Abbreviations in order: Texas Christian University Drug Screen (TCUDS); Substance Use and Abuse Survey (SUAS); South Shore Problem Inventory-revised (SSPI); The Simple Screening Instrument for Substance Abuse (SSI-SA); The Simple Screening Instrument (SSI); Screen of Drug Use (SoDU); Single-Item Screening Questions (SISQs); Medical Outcomes Survey Short-Form 12-Item Health Survey (SF-12); Substance Dependence Severity Scale (SDSS); Substance Abuse Subtle Screening Inventory-2 (SASSI-2); Rapid Opioid Dependence Screen (RODS); Quantity-Frequency Index (QFI); PTSD Checklist–Civilian version (PCL-C); PTSD Checklist 4 Item (PCL-Bliese-4); PTSD Checklist 2 Item (PCL-LS-2); PTSD Checklist 3 Item (PCL-LS-3);PTSD Checklist 4 Item (PCL-LS-4);PTSD Checklist 6 Item (PCL-LS-6);Primary Care–PTSD screen (PC-PTSD); Personality Assessment Inventory Drug Problem Scale (PAI DRG); National Epidemiologic Survey on Alcohol and Related Conditions (NESARC); The Marijuana Screening Inventory (MSI-X); The Longitudinal Substance Use Recall Instrument Recall for 12 Weeks Instrument (LSUR-12); The Longitudinal Substance Use Recall Instrument (LSUR); Lifetime Severity Index for Cocaine Use Disorder (LSI-Cocaine); Healthcare Effectiveness Data and Information Set (HEDIS); Global Appraisal of Individual Needs Quick version 3 (GAIN-03); The Drug Use Screening Inventory (DUSI); Dartmouth Assessment of Lifestyle Instrument (DALI); CJDATS Co-Occurring Disorders Screening Instruments for Severe Mental Disorders (CODSI-SMD); CJDATS Co-Occurring Disorders Screening Instruments for any Mental Disorder (CODSI-MD); Cut down, Annoyed, Guilty, and Eye-Opener Substance Abuse Screening Tool (CAGE); The Alcohol Use Disorder and Associated Disabilities Interview Schedule (AUDADIS); Alcohol, Smoking, and Substance Involvement Screening Test-Drug (ASSIST-Drug); Parents, Partners, Past and Pregnancy Plus (4P’s Plus); Tobacco, Alcohol, Prescription Medication, and Other Substance Use (TAPS Tool); Substance Use Brief Screen (SUBS); Single Question Used from Short Inventory of Problems-Drug Use (SIP-DU);  Drug Abuse Screening Test (DAST); Cannabis Use Identification Test Revised (CUDIT-R); The Chemical Use, Abuse, and Dependence (CUAD); Addiction Severity Index (ASI)
